# Biased signaling due to oligomerization of the G protein-coupled platelet-activating factor receptor

**DOI:** 10.1038/s41467-022-34056-4

**Published:** 2022-10-26

**Authors:** Junke Liu, Hengmin Tang, Chanjuan Xu, Shengnan Zhou, Xunying Zhu, Yuanyuan Li, Laurent Prézeau, Tao Xu, Jean-Philippe Pin, Philippe Rondard, Wei Ji, Jianfeng Liu

**Affiliations:** 1 Cellular Signaling laboratory, International Research Center for Sensory Biology and Technology of MOST, Key Laboratory of Molecular Biophysics of MOE, College of Life Science and Technology, Huazhong University of Science and Technology, 430074 Wuhan, Hubei China; 2 Institut de Génomique Fonctionnelle, Université de Montpellier, CNRS, INSERM, 34094 Montpellier, Cedex France; 3 National Laboratory of Biomacromolecules, CAS Center for Excellence in Biomacromolecules, Institute of Biophysics, Chinese Academy of Sciences, Beijing, China; 4 Guangzhou Regenerative Medicine and Health Guangdong Laboratory, Chinese Academy of Sciences, 510005 Guangzhou, China

**Keywords:** Receptor pharmacology, G protein-coupled receptors

## Abstract

G protein-coupled receptors (GPCRs) are important drug targets that mediate various signaling pathways by activating G proteins and engaging β-arrestin proteins. Despite its importance for the development of therapeutics with fewer side effects, the underlying mechanism that controls the balance between these signaling modes of GPCRs remains largely unclear. Here, we show that assembly into dimers and oligomers can largely influence the signaling mode of the platelet-activating factor receptor (PAFR). Single-particle analysis results show that PAFR can form oligomers at low densities through two possible dimer interfaces. Stabilization of PAFR oligomers through cross-linking increases G protein activity, and decreases β-arrestin recruitment and agonist-induced internalization significantly. Reciprocally, β-arrestin prevents PAFR oligomerization. Our results highlight a mechanism involved in the control of receptor signaling, and thereby provide important insights into the relationship between GPCR oligomerization and downstream signaling.

## Abstract (web summary)

The functional consequence of G protein-coupled receptor oligomerization remains debated. Here the authors show that platelet-activating factor receptor oligomerization enhances G protein coupling, and restrains β-arrestin recruitment and internalization.

## Introduction

G protein-coupled receptors (GPCRs) form the largest family of cell surface receptors. GPCRs are targeted by approximately one-third of all approved drugs^1^. Many GPCRs can activate several intracellular signaling cascades through interactions with various types of G proteins^2^ and β-arrestins^3,4^. Distinct agonists acting on the same GPCR can engage different effector subsets, and modify cellular outcomes differently. This phenomenon is termed “biased signaling”^5,6^. The discovery of biased ligands that favor specific signaling pathways highlights the relevance of precise control of GPCR signaling for proper therapeutic action with fewer side effects^7–10^. Although significant effort has been spent in the past to understand how a ligand can orient GPCR signaling towards one or another pathway^10–13^, less is known about the physiological molecular mechanisms that orient GPCR signaling. For example, proteins that interact with GPCRs or lipid membrane composition are expected to control the balance between GPCR-mediated signaling pathways^12^.

Many GPCRs can assemble into dimers or oligomers^14–17^ even in native tissues^18,19^, and such assemblies were suggested to play important roles in physiological and pathological processes^20,21^. Monomeric class A GPCRs are known to be able to activate multiple G proteins^22,23^ and β-arrestins^24–29^. However, a dynamic equilibrium between monomer and di/oligomer populations has also been observed for several GPCRs^30–32^. Di/oligomerization of class A GPCR receptors may affect their intracellular trafficking^33^ via either positive or negative cooperative ligand binding^18,34^, increase^35,36^ or decrease^37,38^ in G protein activation and modify G protein coupling selectivity^39,40^. However, the role of di/oligomerization of GPCRs in β-arrestin engagement^31^ remains unclear.

The platelet-activating factor (PAF) receptor (PAFR) is the natural receptor for an endogenous lipid, 1-O-alkyl-2-acetyl-sn-glycero-3-phosphocholine (PAF). PAFR is involved in platelet aggregation and inflammatory responses^41^. Following its activation, PAFR couples to both G_q/11_ and G_i/o_ and also possibly to G_12/13_^42^ and recruits β-arrestin1 (βarr1) to initiate receptor internalization^41,43,44^. Furthermore, PAFR plays an important role in many physiological and pathological events, including immune response, cardiovascular regulation, and tumor growth^45–47^. We recently reported the crystal structure of PAFR with antagonists, which included an unusual conformational arrangement of seven transmembranes (7TM) domains^48^. However, the structural basis of PAFR di/oligomer assembly, and its role in controlling signaling remains unclear.

Here, we report the formation of PAFR dimers and oligomers even at low densities in transfected cell lines. Using the cysteine (Cys) cross-linking strategy, we identify two types of symmetric PAFR dimer interfaces, one involving TM1 and the other TM4 and 5. Locking either one or both of these interfaces via Cys cross-linking stabilizes the PAFR dimers and oligomers, respectively. We also demonstrate increased G protein signaling as well as decreased β-arrestin recruitment and agonist-induced PAFR internalization due to the stabilization of PAFR di/oligomers. Such dimerization-induced biased signaling is also observed with a natural genetic variant of PAFR. Our findings thus reveal a mechanism by which GPCRs control signaling bias through oligomerization.

## Results

### PAFR oligomerization on cell membranes

**Fig. 1:**
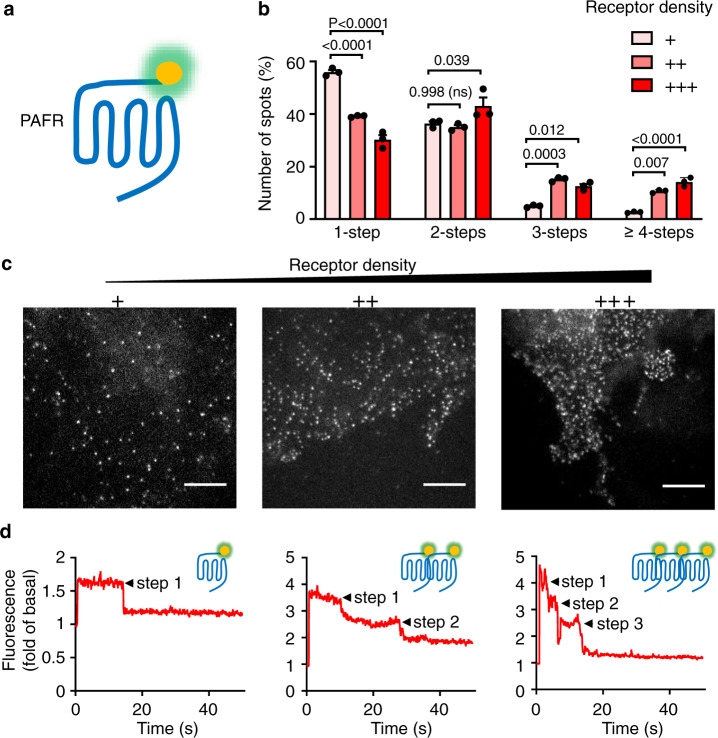
Single-molecule photobleaching analysis shows PAFR oligomer formation on the cell surface. **a** Schematic representation of Halo-tagged PAFR labeled with the fluorescent Halo-488 substrate. **b** Histogram of photobleaching step numbers for all Halo-PAFR molecules analyzed. Cells expressing three different densities of PAFR were used: +: 1994 spots from 21 movies, ++: 1956 spots from 15 movies, +++: 1995 spots from 17 movies. Analyzed spots are from 5 to 7 movies each time and repeated three times. Data are mean ± SEM from *n* = 3 biologically independent experiments and were analyzed using one-way ANOVA with Dunnett’s multiple comparisons test. **c** Dependence of the distribution of monomers and dimers/oligomers on receptor density. +: 0.22 ± 0.07 particles/μm^2^; ++: 0.42 ± 0.07 particles/μm^2^ and +++: 0.76 ± 0.21 particles/μm^2^. Scale bar = 5 µm. Data are representative of a typical experiment from *n* = 3 biologically independent experiments. **d** Representative fluorescence time course for an individual molecule. Left, 1-step photobleaching (monomer); middle, 2-steps photobleaching (dimer) and right, 3-steps photobleaching (trimer). Of note, the photobleaching process was recorded during the entire period, including the period of the very low laser excitation needed to detect the cells and identify the area to be recorded. Afterward, the laser energy was increased to record the signal, generating the upstroke visible shortly after the beginning of the trace.

We first examined the formation of PAFR dimers or oligomers using single-molecule imaging of Halo-tagged receptors covalently labeled with non-cell-permeant fluorophores expressed at various densities (Fig. 1a). We determined that ~80% of the Halo-PAFR were labeled with Halo-Alexa488, using a mCherry-fused Halo-PAFR, where only 80% of the mCherry fluorescent particles were colocalized with the Alexa488 signal (Supplementary Fig. 1a–c). Images of individual PAFRs were obtained by TIRF microscopy, and photobleaching step analysis was performed (Fig. 1b–d). As a control, measurements with Halo-tagged obligatory dimeric metabotropic glutamate (mGlu) receptors (Halo-mGlu2) were performed. For this mGlu2 receptor, we observed ~60% two-step events at a density of 0.39 ± 0.01 spots/μm^2^ (Supplementary Fig. 2a–d), in agreement with literature^49^. This is also in agreement with the 80% efficacy of labeling of the Halo-tag for each subunit in the mGlu2 dimer where 0.8 × 0.8 (equivalent to 64%) of the dimers are labeled on both subunits.

When performed with the PAFR, a mixture of monomers and di/oligomers were detected, and showed a density-dependent di/oligomerization behavior^50,51^ (Fig. 1b). When more receptors were expressed on cell membranes, the one-step photobleaching ratio was reduced from ~56% (+: 0.22 ± 0.07 particles/μm^2^) to ~30% (+++: 0.76 ± 0.21 particles/μm^2^). Since the labeling efficiency of the fluorescent probe is about 80%, mono/di/oligomeric fraction calculated from the photobleaching step required further correction. After correction, as described in the “Methods” section, for the low expression level of the PAFR, a high percentage of particles corresponds to monomers and dimers (43.5% monomers and 46.7% dimers). But at high density, 56.0% correspond to dimers and 30.7% to oligomers (13.5% trimers and 17.2% tetramers), with a monomer fraction at 13.3%. We can also estimate that at low density, 25% of the PAFR receptor entities are in a monomeric state, while 55% of them are part of dimers. At high density, only 5% of the PAFR entities are in a monomeric state, while 46% of them are in a dimer, and 49% are part of oligomers (*n* ≥ 3).

In these experimental conditions, the quantity of receptors (*B*_max_) is 7.0 and 26.5 pmol/mg of total proteins, respectively (Supplementary Fig. 3a, b). It corresponds to a density of ~260 and ~900 receptors per cell for low and high density, respectively (assuming a surface area of a COS-7 cell at ~1200 μm^2,^^52^). In the low expression conditions (+), the mean density of receptors is about 2–14-fold higher than the physiological one, as measured by the endogenous expression of PAFR in a mesangial cell line (3.06 pmol/mg)^53^ or in hepatocyte membranes (0.5 pmol/mg)^54^. However, the exact receptor density within the specific membrane sub-compartment of these cells is expected to be higher.

Titration experiments were performed using bioluminescence resonance energy transfer (BRET) technology. The energy donor Renilla luciferase 8 (Rluc) and acceptor yellow fluorescent protein Venus were fused to PAFR C-terminus. A saturation curve with a high maximum BRET ratio was observed for PAFR-Rluc and PAFR-Venus, whereas a lower ratio was obtained for PAFR-Rluc and β_2_AR-Venus (Supplementary Fig. 4a). Furthermore, competition BRET experiments were performed with different numbers of untagged receptors (i.e. without Rluc or Venus tags), which are expected to decrease in the BRET signal via competition with tagged receptors for dimerization. Indeed, co-expression of untagged PAFR yielded a gradual and linear decrease in the BRET signal (Supplementary Fig. 4b), whereas co-expression of β_2_AR showed a modest effect possibly resulting from the capacity of this receptor to interfere with the formation of PAFRs only at high expression levels (Supplementary Fig. 4c). Similar expression levels of PAFR and β_2_AR were observed due to the N-terminal Flag epitope (Supplementary Fig. 4d). In summary, these results indicated density-dependent PAFR oligomerization on the cell membrane.

### Characterization of the PAFR 7TM dimer interface

**Fig. 2:**
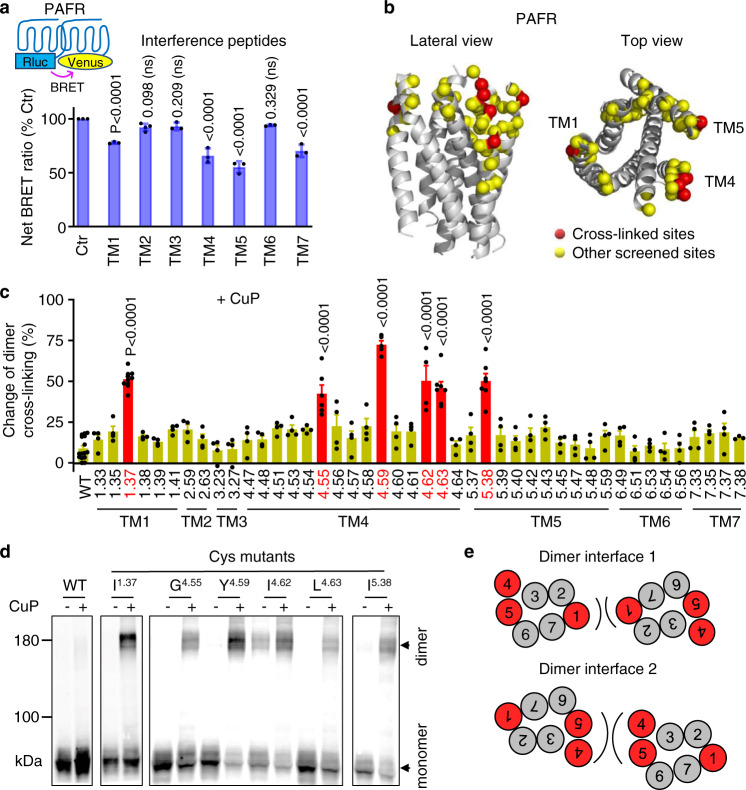
Cysteine cross-linking identifies TM1, TM4, and TM5 at the PAFR dimer interface. **a** Effects of interference peptides derived from the indicated transmembrane domains on the BRET signal between PAFR molecules. BRET signal was measured from cells co-transfected with PAFR-Venus and PAFR-Rluc after incubating with 10 μM of the indicated peptide for 2 h. Data are mean ± SEM from *n* = 3–4 biologically independent experiments performed in triplicates and were analyzed using one-way ANOVA with Dunnett’s multiple comparisons test, compared with control (Ctr, pure DMSO). **b** 3D structure of the 7TM of PAFR (PDB code: 5ZKQ) with all Cys substitutions highlighted either by a yellow sphere (α carbon) or by a red sphere for those well cross-linked in TM1, TM4, and TM5. **c** Change of PAFR dimer rate induced by CuP treatment for the WT and the indicated mutants. Representative blots are shown in Supplementary Fig. 6. Positions with a significant change were highlighted in red. Data are mean ± SEM from *n* = 3–7 biologically independent experiments and were analyzed using one-way ANOVA with Dunnett’s multiple comparisons test. Statistical analysis not significant is not shown. **d** Cross-linking of the indicated cell surface Halo-PAFR subunits labeled with fluorescent Halo substrates, after treatment (+) or without treatment (−) with CuP. After SDS–PAGE in nonreducing conditions, Halo-PAFR monomers and dimers were detected via the fluorophore covalently attached to the receptors. Data are representative of a typical experiment from *n* = 4–7 biologically independent experiments. **e** Dimerization interface based on the results of the cross-linking experiments. TMs that can cross-link between protomers are highlighted in red.

Peptides with sequences corresponding to transmembrane helices can disrupt receptor–receptor interactions in GPCRs^55^ as they form part of the protein–protein interaction interface. We thus used such peptides and performed a BRET assay to identify possible dimerization interfaces on the 7TMs of PAFR. The addition of the synthetic peptides TM1, TM4, TM5, and TM7 decreased the BRET signal between PAFR-Rluc and PAFR-Venus, whereas the remaining three peptides did not show significant effects (Fig. 2a). These results also suggest the existence of PAFR di/oligomers in cell membranes, and multiple potential dimeric interfaces composed of TM1, TM4, TM5, and TM7. But these results need to be confirmed by another method since the correct folding of such peptides cannot be ascertained.

We then used a Cys cross-linking strategy to further characterize the dimeric interfaces. Cys cross-linking provides a better resolution of the proximity between two residues in protein–protein interactions (Fig. 2b) as it requires a distance of less than 8 Å between the Cβ of both cysteines. Analysis of the Cys cross-linked Halo-tagged PAFR that was only localized at the cell surface was possible due to the use of non-cell-permeant fluorescent Halo-tag substrates to label the living cells before blot analysis. We used this approach previously to identify the dynamic interfaces of three class C GPCRs: mGlu2^56^, γ-amino-butyric acid GABA_B_^57^, and calcium-sensing (CaS) receptors^58^. Treatment with oxidative copper phenanthroline (CuP) was used to promote the cross-linking of two cysteines, which can be detected through the appearance of dimers by SDS–PAGE and protein transfer to membranes^57,58^.

Using this approach, we screened 44 amino acid positions by replacing each respective residue in each TM with cysteine one by one, as illustrated in Fig. 2b and Supplementary Fig. 5. Similarly to the reported studies^56–58^, we mainly considered receptor–receptor interaction within the extracellular part of the transmembrane helices, as this part is more prone to be oxidized, the intracellular compartment being reducing. We also quantified the change in the ratio of dimers to the total quantity of PAFR subunits detected on the blots to determine the efficiency of CuP-induced cross-linking between the two monomers (Fig. 2c and Supplementary Fig. 6). These experiments revealed efficient cross-linking of PAFRs when cysteines were introduced into TM1, TM4, and TM5 (Fig. 2c, d). However, no such cross-linking was observed when cysteines were introduced into TM2, TM3, TM6, or TM7 (Fig. 2c and Supplementary Fig. 6a). No significant CuP-induced cross-linking was observed for the wild-type (WT) receptor. A PAFR antagonist (ABT-491^59^) was used to enhance cell surface expression^60^ due to low expression levels of several cysteine mutants (Supplementary Fig. 6b). Altogether, our data revealed two possible PAFR dimer interfaces: one involving TM1, and the other involving TM4 and TM5 (Fig. 2e).

### Stabilization of PAFR oligomers via cysteine cross-linking

**Fig. 3:**
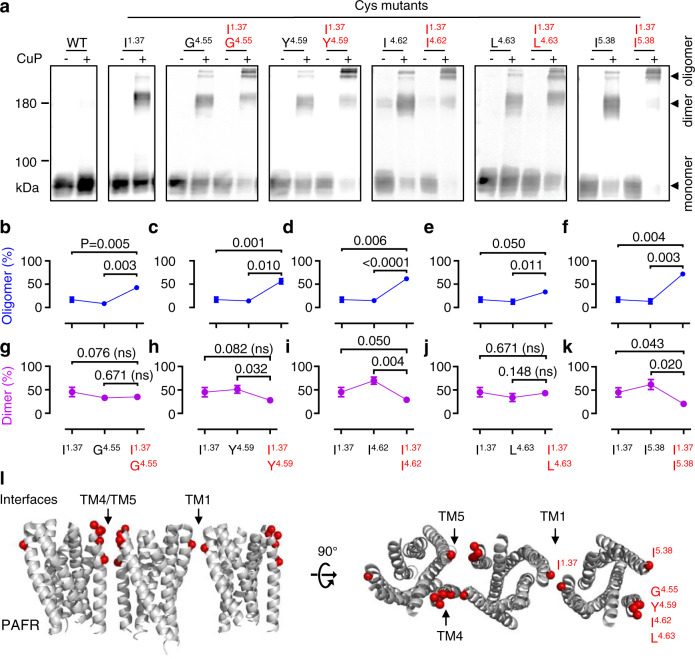
Double cross-linking at TM1–TM4 or TM1–TM5 contributes to higher-order PAFR oligomers. **a** Cross-linking of the indicated subunits containing Cys substitutions in TM1, TM4, and TM5 after treatment (+) or without treatment (−) with CuP. Data are representative of a typical experiment from *n* = 3 biologically independent experiments. **b–k** Dimer/oligomer ratio of the indicated mutants relative to the total amount of PAFR was quantified from panel **a** (+CuP). Data are mean ± SEM from *n* = 3 biologically independent experiments and were analyzed using paired two-tailed *t*-test. **l** 3D model of a PAFR oligomer (three protomers are shown) with the TM1 and TM4/TM5 interfaces highlighted.

We stabilized PAFR oligomers by Cys cross-linking at TM1 and TM4/5 dimer interfaces which belong to two opposite faces of 7TM (Fig. 3a). The cysteines that cross-linked well at these dimer interfaces were combined in the same protomer of the receptor. Next, we tested a series of double cysteine mutations in TM1–TM4 or TM1–TM5 (I20C^1.37^-G147C^4.55^, I20C^1.37^-Y151C^4.59^, I20C^1.37^-I154C^4.62^, I20C^1.37^-L155C^4.63^, and I20C^1.37^-I186C^5.38^; see nomenclature of class A GPCRs^61^). As expected, the presence of CuP resulted in a significant increase in the oligomer ratio of all double cysteine mutants (Fig. 3b–f). Of note, the amount of dimer fraction was reduced or did not change for the double cysteine mutants when compared to the single cysteine mutants, most likely because they could further associate into oligomers (Fig. 3g–k). Hence, monomers or dimers are organized into oligomers via double cysteine cross-linking. These findings also support the existence of possible PAFR oligomers composed of two symmetrical interfaces (Fig. 3l).

### PAFR oligomers distinctly modulated G protein activation and β-arrestin recruitment

**Fig. 4:**
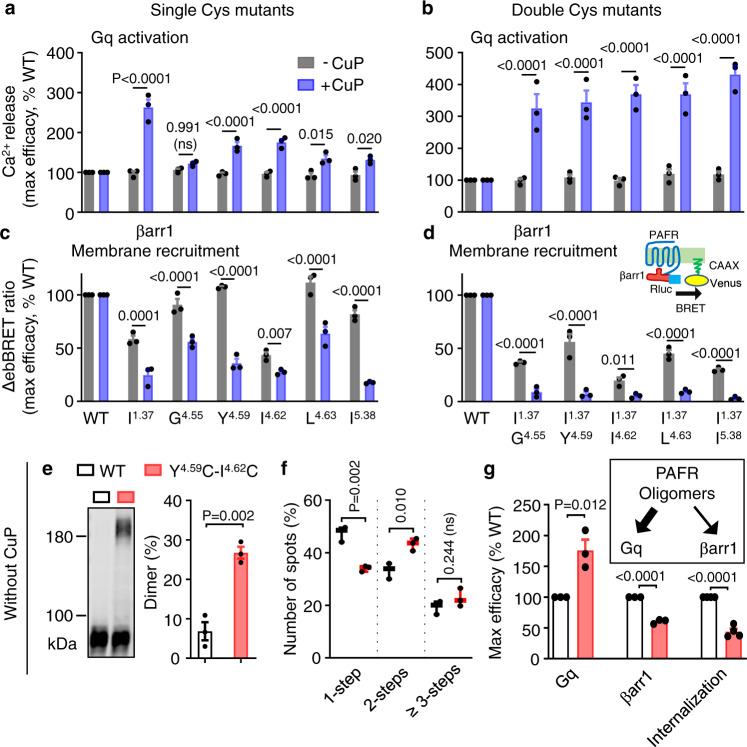
PAFR oligomers distinctly modulate G protein activation and β-arrestin recruitment. **a**, **b** Intracellular Ca^2+^ responses mediated by the indicated single and double Cys substitutions in the indicated TM upon stimulation with PAF. The indicated maximal efficacy is from the dose–response in Supplementary Fig. 7b, c. **c**, **d** Recruitment of βarr1 to the cell membrane by the indicated mutants upon stimulation with PAF (1 μM) and monitored by the variation of ebBRET signal between βarr1-Rluc and Venus-CAAX. The indicated maximal efficacy is from Supplementary Fig. 8b, c. Data in panels **a**–**d** are mean ± SEM from *n* = 3 biologically independent experiments and were analyzed using one-way ANOVA with Dunnett’s multiple comparisons test. **e** Blots showing cross-linking of the cell surface Halo-PAFR subunits containing a double cysteine substitution in TM4 (Y151C^4.59^-I154C^4.62^) as indicated without treatment with CuP. Data are mean ± SEM of *n* = 3 biologically independent experiments and were analyzed using paired two-tailed *t*-test. The blot is representative of a typical experiment from *n* = 3 biologically independent experiments. **f** Photobleaching step analysis for the WT and the mutant Y151C^4.59^-I154C^4.62^ with spots analyzed from more than six movies by experiment and repeated three times: 1945 spots from 29 movies for the WT, and 1561 spots from 17 movies for the mutant. Data are mean ± SEM of *n* = 3 biologically independent experiments. **g** Maximal efficacy of Gq activation, βarr1 recruitment, and agonist-induced internalization for the WT and the mutant Y151C^4.59^-I154C^4.62^ from experiments shown in Supplementary Fig. 10d, e, and g. Data are mean ± SEM of *n* = 3–4 biologically independent experiments performed in triplicates and normalized to WT. Data in panels **f** and **g** were analyzed using an unpaired two-tailed *t*-test.

We next investigated the effect of PAFR oligomerization on its transducers by measuring levels of Gq activation, βarr1 recruitment, and receptor internalization^41,43,44^. For this purpose, we used PAFR mutants forming either stable dimers with a single cysteine mutation in TM1, TM4, and TM5 (Fig. 2c) or stable oligomers with double cysteine mutations in TM1 and TM4/5 (Fig. 3). After CuP treatment, these PAFR mutants had higher Gq signaling efficacies upon agonist stimulation (Fig. 4a, b and Supplementary Fig. 7a–c) with similar cell surface expression levels (Supplementary Fig. 7d). In the absence of CuP treatment, however, the mutants showed activity similar to that of the WT receptor, indicating that the mutations did not affect receptor signaling per se (Fig. 4a, b and Supplementary Fig. 7c). Hence, PAFR oligomerization increased Gq signaling.

We then investigated βarr1 recruitment to PAFR mutants using an enhanced bystander BRET (ebBRET) assay^62^ (Supplementary Fig. 8a). An increased BRET signal between βarr1-Rluc and the membrane-anchored Venus-CAAX was measured upon agonist stimulation of both WT and mutant PAFR (Fig. 4c, d and Supplementary Fig. 8b, c). However, βarr1 recruitment to these mutants decreased significantly after CuP treatment (Fig. 4c, d and Supplementary Fig. 8b), with no agonist recruitment for the double cysteine mutants. Similar results were obtained using another BRET assay between βarr1-Rluc and PAFR-Venus (Supplementary Fig. 9a–d) for both WT and mutants that are well-expressed at the cell surface expression levels (Supplementary Fig. 9e). Di/oligomerization of PAFR thus decreased βarr1 recruitment.

These cross-linking experiments required copper treatment that could induce cell toxicity and disrupt PAFR signaling, as shown by the absence of PAFR internalization after CuP treatment (Supplementary Fig. 10a, b). Therefore, we searched for mutations that favored oligomer formation in the absence of CuP as well. Among the 10 double cysteine mutants in TM4 and TM5 (Supplementary Fig. 10c), we found that the double mutant Y151C^4.59^-I154C^4.62^ in TM4 could be cross-linked without CuP treatment (Fig. 4e). This is in agreement with the tendency of this double mutant to form a high proportion of dimers and oligomers as observed in the photobleaching analysis (Fig. 4f). Finally, this mutant also showed higher Gq efficacy (~175% of WT), and lower βarr1 recruitment (~60% of WT) (Fig. 4g and Supplementary Fig. 10d, e) at an expression level similar to that of the WT (Supplementary Fig. 10f).

This double mutant also impaired PAFR internalization significantly (~45% of WT) as evidenced by results of a diffusion-enhanced resonance energy transfer (DERET) internalization assay^63^ (Fig. 4g and Supplementary Fig. 10g). This is in agreement with the internalization of PAFR WT, which is βarr-dependent (Supplementary Fig. 10h). Indeed, no agonist-induced internalization of the receptor was measured in HEK-293 cell KO for both βarr1 or βarr2 (Supplementary Fig. 10h). However, agonist-induced internalization was rescued by transfection with βarr1, in agreement with agonist-induced internalization of the receptor in the parental cells.

### A natural genetic variant of PAFR promoted dimerization and Gq-biased signaling

**Fig. 5:**
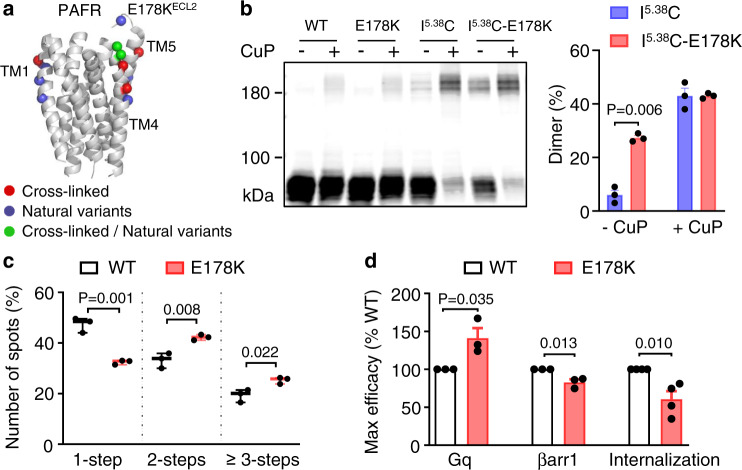
A natural genetic variant promotes PAFR dimerization and induces biased signaling. **a** 3D structure of the 7TM of PAFR (PDB code: 5ZKQ), lateral view. Amino acids (α carbon) that can be cross-linked by a cysteine in TM1, 4, and 5 (red sphere), that corresponds to natural genetic variants (blue sphere), and that is in both situations (green sphere) are highlighted. **b** Blots showing cross-linking of cell surface Halo-PAFR subunits containing a cysteine substitution in TM5 (I186C^5.38^) or containing the indicated variant together with I186C^5.38^, with or without treatment with CuP. Data are mean ± SEM of *n* = 3 biologically independent experiments and were analyzed using paired two-tailed *t*-test. The blot is representative of a typical experiment from *n* = 3 biologically independent experiments. **c** Summary of photobleaching step analysis for the WT and indicated PAFR variant where the spots are analyzed from more than five movies for one experiment and repeated three times: 1945 spots from 29 movies for the WT, and 1686 spots from 15 movies for the variant. Data are mean ± SEM of *n* = 3 biologically independent experiments and were analyzed using an unpaired two-tailed *t*-test. **d** Maximal efficacy for Gq activation, βarr1 recruitment, and agonist-induced internalization of the WT and PARFR variant from experiments shown in Supplementary Fig. 12. Data are mean ± SEM from *n* = 3–4 biologically independent experiments performed in triplicates and normalized to WT, and were analyzed using an unpaired two-tailed *t*-test.

Engineered^64^ and genetic mutations^65^ were previously shown to control GPCR dimer formation, and are therefore highly relevant for diseases. For example, mutations impairing rhodopsin dimerization have been found in retinitis pigmentosa^65^. We thus investigated whether natural genetic variation could also influence PAFR oligomerization^66^ (Fig. 5a).

For this purpose, we introduced residue substitutions into dimer-producing single cysteine mutants to identify mutations that favor PAFR dimer formation (Fig. 5b and Supplementary Fig. 11a–c). We found that the variant with E178K located in the extracellular loop 2 (ECL2) displayed a larger proportion of I186C^5.38^ dimers in the absence of CuP, and thus a higher dimer formation probability. This finding was also validated by the photobleaching step analysis of single particles (Fig. 5c). Consistent with our previous findings, PAFR-E178K showed higher Gq signaling (~141% of WT) and lower βarr1 recruitment (~83% of WT) and agonist-induced internalization (~69% of WT) efficacies than the WT receptor (Fig. 5d and Supplementary Fig. 12a–c), at similar expression levels as that of the WT receptor (Supplementary Fig. 12d, e).

### Increased Gq coupling of PAFR dimers is not only due to the lack of βarr recruitment

**Fig. 6:**
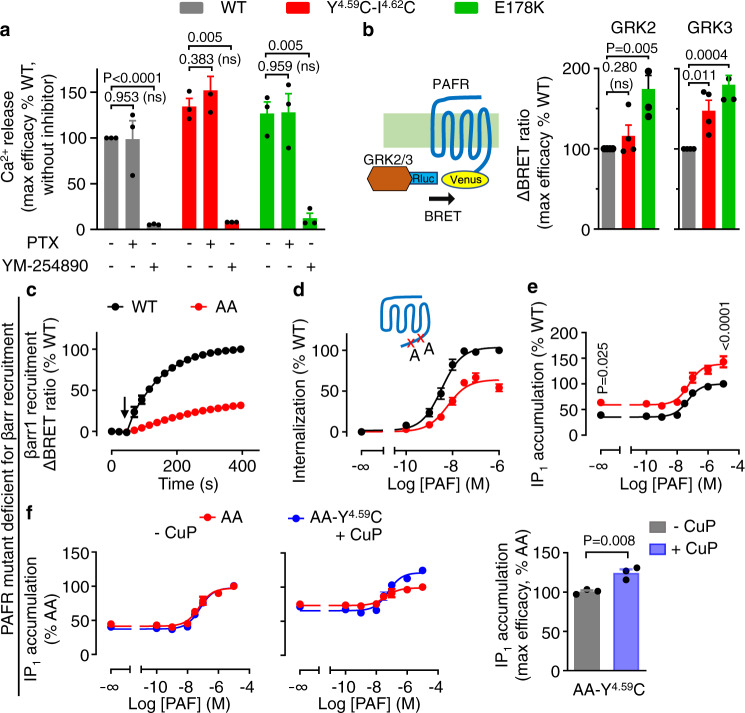
The increase of G protein coupling is not only due to the lack of βarr recruitment. **a** Intracellular Ca^2+^ responses mediated by the indicated PAF receptors upon stimulation with PAF (1 μM), after or without treatment with PTX (100 ng/ml) or YM-254890 (100 nM). The indicated maximal efficacy is from the dose–response in Supplementary Fig. 13a. **b** Recruitment of GRK2-Rluc or GRK3-Rluc to the Flag-tagged PAFR-Venus upon stimulation with PAF (1 μM). The indicated maximal efficacy is from Supplementary Fig. 14a and d. **c** Kinetics of the BRET signal between PAFR-Venus and βarr1-Rluc after the injection of PAF (1 μM, arrow) for the PAFR WT and indicated AA mutant (T324A, E327A; scheme in panel **d**). **d** Agonist-induced internalization of the AA mutant and WT PAFR. **e** IP_1_ accumulation mediated by the PAFR WT and AA mutant upon stimulation with PAF. **f** IP_1_ accumulation mediated by the indicated PAFR AA mutants, with or without the mutation Y^4.59^C, upon stimulation with PAF, after or without treatment with CuP. Data in **a***–***f** are mean ± SEM from *n* = 3–4 biologically independent experiments performed in triplicates and normalized to indicated conditions and analyzed using unpaired two-tailed *t*-test.

We further investigate the molecular mechanism of the G protein-biased signaling observed above for the receptor dimers/oligomers. First, we confirm that the PLC-mediated intracellular calcium releases measured are through Gq activation and not Gi. Indeed, Gi/o proteins can either directly activate PLC through their Gβγ subunit^67^, or boost Gq-mediated PLC activation, as observed in many studies^68^. We then examined the effect of the Gi/o inhibitor pertussis toxin (PTX), and the Gq inhibitor YM-254980^69^. YM-254980, but not PTX, totally suppressed the calcium response mediated by the WT, the mutant Y151C^4.59^-I154C^4.62^ and the variant E178K, demonstrating the Gq activation is needed for the calcium release effect (Fig. 6a and Supplementary Fig. 13a, b). Of note, under the same conditions, the PAFR-mediated inhibition of cAMP formation measured with the EPAC sensor is suppressed after PTX treatment (Supplementary Fig. 13c), demonstrating the Gi/o pathway has been correctly inhibited under our conditions.

Second, for most class A GPCRs, βarr recruitment follows receptor phosphorylation by the G protein-coupled receptor kinase (GRKs)^4^. It is then possible that the absence of βarr recruitment to PAFR dimers results from the lack of GRKs recruitment and phosphorylation of the receptor. We show, using a BRET approach, that the PAFR-Venus can recruit the Rluc-tagged GRK2 or GRK3 upon activation, for either the WT, the double mutant Y151C^4.59^-I154C^4.62^ or the E178K variant that spontaneously form dimers (Fig. 6b and Supplementary Fig. 14a, d). GRK2 and GRK3 recruitment could also be observed with the single cysteine mutants in TM4 and 5 that can be crosslinked upon CuP treatment (Supplementary Fig. 14b, c and e, f). These data then show that the lack of βarr recruitment is not the consequence of a lack of GRKs recruitment by the PAFR dimers.

Third, we examined whether the increase in Gq signaling may come from the absence of βarr competition with the G protein binding site on the activated receptor. We first tested the Gq coupling properties of a PAFR mutant deficient for βarr recruitment. This mutant carries two Ser to Ala mutations (T324A, E327A) within the βarr phosphorylation bar code in the C-terminal region of PAFR. This AA mutant is expressed similarly to the WT (Supplementary Fig. 15), but its recruitment of βarr and its internalization is largely inhibited (Fig. 6c, d). Despite a small increase in the efficacy of this PAFR mutant to produce IP_1_ accumulation (Fig. 6e), the efficacy of the Y151C^4.59^ cross-linked dimer is further increased (Fig. 6f). Taken together, the data are consistent with the increased IP_1_ production of the cross-linked dimers being not only the consequence of the lack of βarr recruitment. This is also consistent with our observation that the Gq-mediated Ca^2+^ signal, which occurs within seconds after receptor activation, then much before any significant βarr recruitment, is also largely potentiated by dimer cross-linking (Fig. 4a, b, Supplementary Figs. 7a, b, 10d, and 12a).

**Fig. 7:**
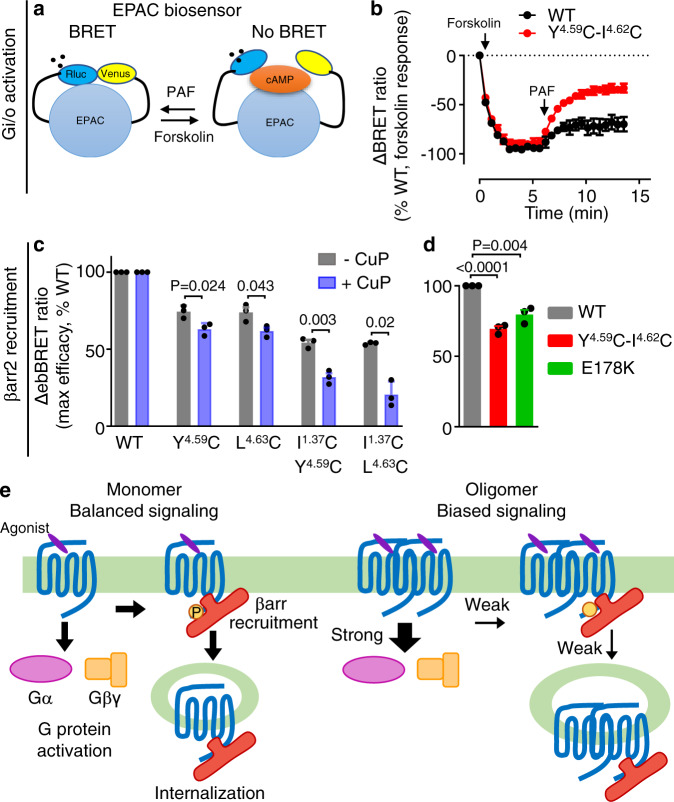
Biased signaling modulation by PAFR oligomerization. **a** Scheme illustrating the BRET assay to measure the cAMP by the exchange protein directly activated by cAMP (EPAC) sensor in HEK-293 cells. **b** Monitoring of cAMP mediated by the WT Flag-tagged PAFR and the mutant. Forskolin (10 μM) and PAF (5 μM) were used to stimulate the cells. Data are mean ± SEM from *n* = 3 biologically independent experiments performed in triplicates and normalized to WT. **c**, **d** Recruitment of βarr2 to the cell membrane by the PAFR WT and indicated single or double mutants upon stimulation with PAF (1 μM) and monitored by the variation of ebBRET signal between βarr2-Rluc and Venus-CAAX. The indicated maximal efficacy is from Supplementary Fig. 16. Data in **c** and **d** are mean ± SEM from *n* = 3 biologically independent experiments performed in triplicates and normalized to WT and analyzed using an unpaired two-tailed *t*-test. **e** Scheme illustrating biased signaling modulation by PAFR oligomerization. PAFR monomers are efficient to couple to G protein and for βarr recruitment (balanced signaling). PAFR oligomers increase G protein coupling efficacy but decrease βarr recruitment and agonist-induced internalization (biased signaling).

Fourth, we verified that the G protein bias is also true for other G proteins and for βarr2. PAFR couples to both G_q/11_ and G_i/o_ and also possibly to G_12/13_^42^. We did also examine the influence of PAFR dimer formation on the inhibition of cAMP formation using the EPAC BRET sensor (Fig. 7a). A stronger inhibition of forskolin-induced cAMP formation was observed with the double mutant Y151C^4.59^-I154C^4.62^, supporting an increase in Gi-protein-mediated effects of the PAFR dimer (Fig. 7b). Finally, we examined the effect of PAFR dimerization on βarr2 recruitment to the plasma membrane, and found data similar to those obtained with βarr1 (Fig. 7c, d and Supplementary Fig. 16).

Based on these findings, we propose a model in which PAFR oligomerization modulates G protein activation and βarr engagement (Fig. 7e). In this model, receptor oligomers increase G protein signaling while decreasing βarr recruitment and agonist-induced receptor internalization.

### β-arrestin impaired constitutive activity and receptor oligomer formation

**Fig. 8:**
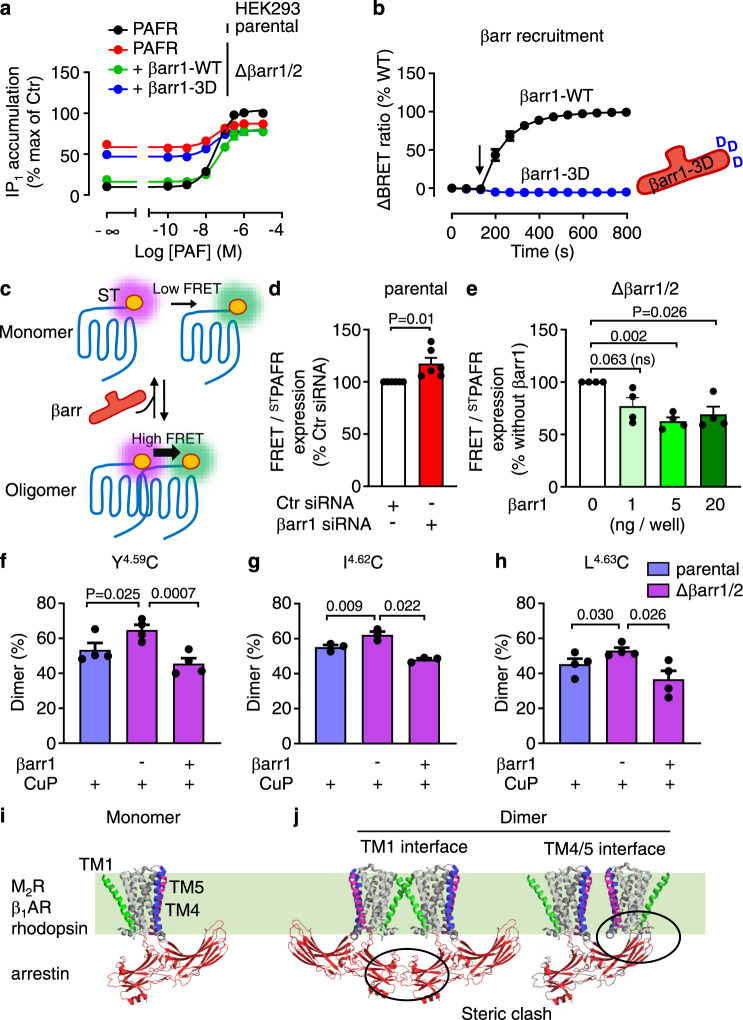
β-arrestin impairs both constitutive activity and PAFR oligomers. **a** IP_1_ mediated by PAFR upon stimulation with PAF in parental (Ctr) or Δβarr1/2 HEK-293 cells. Data are mean ± SEM from *n* = 3 independent experiments and normalized to Ctr. **b** Kinetics of the BRET signal between PAFR-Venus and βarr1-Rluc after the injection of PAF (1 μM, arrow) for the WT and the indicated βarr1-3D mutant (L335D, L338D, S340D). Data are mean ± SEM from *n* = 3 independent experiments and normalized to WT. **c** Scheme illustrating the equilibrium between SNAP(ST)-tagged PAFR monomers and oligomers at the cell surface that is modulated by βarr. Cell surface oligomers are measured by the high TR-FRET signal between the N-terminal SNAP-tag labeled with a pair of non-cell permeant fluorophores compatible with FRET. **d** TR-FRET signal for the SNAP-tagged PAFR WT (^ST^PAFR) corrected by the amount of ^ST^PAFR at the cell surface measured by the emission of non-cell permeant ST-Lumi4-Tb, in parental HEK-293 cells transfected with the indicated siRNA. Data are mean ± SEM from *n* = 6 independent experiments and normalized to control (Ctr) siRNA. **e** TR-FRET signal measurement with Δβarr1/2 HEK-293 cells co-transfected with PAFR and different amounts of plasmid encoding for βarr1 as indicated. Data are mean ± SEM from *n* = 4 independent experiments and normalized to the conditions without βarr1. **f–h** Dimer ratio of blots showing cross-linking of cell surface Halo-PAFR subunits containing one cysteine substitution in TM4 as indicated, after treatment with CuP, in parental or Δβarr1/2 HEK-293 cells. Representative blots are shown in Supplementary Fig. 18. Data are mean ± SEM of *n* = 3–4 independent experiments. Data are analyzed using an unpaired two-tailed *t*-test for **d** and **e**, and a paired two-tailed *t*-test for (**f**–**h**). **i** 3D structure of the muscarinic M_2_ receptor (PDB 6U1N) in complex with βarr1. Similar complexes have been solved for the β_1_-adrenergic receptor (PDB 6TKO) with βarr1 and rhodopsin with arrestin-1 (PDB 5W0P). **j** 3D model of a dimeric structure of the M2 receptor and βarr1 (PDB 6U1N) that interact through the TM1 or TM4/5 interfaces, revealing a steric clash between the two arrestins bound to the receptor. Only one molecule of arrestin is shown for the TM4/5 interface due to a large steric clash.

We further investigate the effect of βarr on G protein activation using βarr1/2 knockout (KO) HEK-293 cells, compared to the parental HEK-293 cells. We verified that these KO cells expressed neither βarr1 nor βarr2 (Supplementary Fig. 17a, b). Interestingly, we measured a higher constitutive Gq activity of the PAFR WT (Fig. 8a), the mutant Y151C^4.59^-I154C^4.62^ and the variant E178K PAFR (Supplementary Fig. 17d) in these βarr1/2 KO HEK-293 cells than in the parental cells (Supplementary Fig. 17c). This constitutive Gq activity was inhibited by overexpressed WT βarr1 (Fig. 8a), but not by a mutated βarr1 that could not be recruited by the receptors (Fig. 8b)^26^. As a control, we observed similar FLAG-tagged receptor expression levels under these conditions (Supplementary Fig. 17e). These data also explain why intracellular calcium release cannot be observed in βarr1/2 KO HEK-293 cells with the PAFR WT, the constitutive activity of the receptor inducing emptying of the calcium stores (Supplementary Fig. 17f).

Our results suggest that oligomerization is less favorable for βarr recruitment, as stabilized PAFR dimers/oligomers recruited less βarr compared to the WT receptor. Reciprocally, we found that βarr affected the number of PAFR oligomers, as revealed by a time-resolved FRET-based assay that enables the measurement of SNAP-tagged PAFR dimerization/oligomerization (Fig. 8c). Using siRNA, we observed that the downregulation of βarr (Supplementary Fig. 17g) significantly increased the FRET signal (Fig. 8d), whereas overexpression of βarr1 in βarr1/2 KO cells decreased this signal (Fig. 8e). This conclusion was further supported by the increased proportion of cross-linked dimers of the cysteine mutants at TM4 (Y151C^4.59^, I154C^4.62^, and L155C^4.63^) in βarr1/2 KO cells exclusively, which can be reduced by overexpression of βarr1 (Fig. 8f–h and Supplementary Fig. 18). Taken together, these results are consistent with the observed effect of the βarr proteins on PAFR oligomer formation.

## Discussion

Like many other GPCRs, PAFR both activates G proteins and recruits βarr. Here, we showed that PAFR forms dimers and oligomers at low expression levels. Based on our recently solved PAFR structure^48^ and a Cys-crosslinking approach, we identified two possible dimer interfaces in PAFR. We further showed that stabilized PAFR dimers and oligomers largely favor G protein signaling over βarr interactions. The higher G protein efficacy is likely due to the conformation of the dimers and oligomers, and probably to a small extent to the βarr proteins that are less recruited to the receptor. Conversely, βarr was found to limit oligomer formation. PAFR oligomerization was thus found to be a mechanism that tunes the balance between different pathways of this receptor.

Since the discovery that GPCRs can activate various signaling pathways, it is of interest to understand the mechanisms, if any, that can control the balance between these different pathways. The discovery of biased ligands, i.e. ligands that promote one pathway over another, may allow us to better understand the molecular basis of pathway preferences^12,70^. Among other possibilities^11,71,72^, distinct GPCR conformations stabilized by biased ligands and likely with different affinities for G proteins and arrestins were proposed to activate one or another signaling cascade. This has also been demonstrated through a structural analysis of class A GPCRs where residues interacting specifically with biased ligands were identified^73–75^. These residues are often those that form the agonist-binding pocket and are involved in interhelical interactions as well. Biased ligands induce slightly different positions in the intracellular part of TMs, in particular TM5, TM6, and TM7^28,73,75^. Since TM5 is often involved in dimer formation, its positioning may be affected by dimerization. This may thus indicate a possible link between dimerization and signaling bias.

In a cellular context, several factors can influence the ability of a GPCR to activate a particular signaling pathway^10,12,76^. These factors include post-translational modifications of the receptor^77^, membrane lipids such as cholesterol^23,78^, endogenous ions^79^, level of expression of the transducers G proteins and βarrs, and the interactions between these transducers^80,81^. Subcellular localization of the receptor can also affect the receptor signaling bias^82^. Interestingly, most of these factors also affect the formation of GPCR dimers, as evidenced by cholesterol-induced dimerization^83^, or differences in receptor density and dimer proportions between various intracellular compartments^82^.

The effect of dimerization on the signaling of the complex has been studied mostly based on hetero-oligomers^84^. The effect of homodimerization on biased signaling has not been clearly reported, mostly due to the difficulty in stabilizing dimers in a cellular context. This technical difficulty was overcome through Cys-cross-linking here. Cys crosslinking is also easier to control than other chemical cross-linking strategies used previously^85^. The receptors for which the effect of homo-dimerization or homo-oligomerization was examined on G protein coupling do not provide a clear view of what could happen in most GPCRs. Indeed, when using purified receptors, GPCR dimerization was previously reported to decrease G protein coupling efficacy^37,38^, supporting the view that in a GPCR dimer, only one protomer is active^86–88^. In class C dimeric GPCRs, only one protomer at a time is considered to activate G proteins^87,89–92^ and oligomerization further decreases G protein coupling efficacy^93,94^. However, for class A GPCRs in mammalian cells^36^ and yeast^95^, oligomerization was suggested to increase G protein signaling. Nevertheless, other signaling pathways were not considered in these studies. Hence, the influence of dimerization on receptor signaling bias remains unclear.

In the case of PAFR, we showed that oligomerization largely favored G protein signaling over βarr recruitment and receptor internalization. The increase of agonist-induced G protein coupling might have two components. First, the PAFR dimers/oligomers could promote a favorable conformation for the active state of the G protein. Alternatively, since these dimers/oligomers impair βarr recruitment, it could result in a higher G protein coupling due to less competition with βarr to interact with PAFR. Our data strongly suggest that higher G protein coupling efficacy does not mainly result from this lack of βarr recruitment. Indeed, in the HEK-293 cells, a similar higher agonist-induced G protein coupling was observed for the crosslinked PAFR mutant deficient for βarr recruitment (Fig. 6f), the cross-linked dimers/oligomers (Fig. 4a, b and g) and the genetic variant (Fig. 5d).

The molecular basis for the lack of recruitment of βarr to the PAFR dimers and oligomers could be due to a specific conformation of the dimers/oligomers stabilized by the cross-linking that does not allow the dynamic flexibility to engage βarr. Alternatively, it could be due to the dimeric or oligomeric form of the receptor, and possible steric hindrance resulting from the formation of PAFR dimers, even though the stoichiometry 2:1 for the interaction between rhodopsin and arrestin has been proposed^96^, together with the stoichiometry 1:1. At the same time, our hypothesis is based on receptor:arrestin structures of other receptors that have revealed two different orientations of arrestin relative to the receptor^24–28^. Accordingly, the C-edge loop of arrestins is inserted into the membrane near either TM4/5^24,26,28^, or TM2/3^25,27^ (Fig. 8i and Supplementary Fig. 19). In our study, βarr was found to be oriented towards the TM4/5 dimer interface, providing an explanation for why it prevents dimer formation. It was also found not compatible with the formation of dimers through the TM1 interface (Fig. 8j). This could explain why PAFR oligomers are not prone to recruit βarr1. Notably, no such clashes were observed when arrestin interacted in another orientation (Supplementary Fig. 19), allowing βarr recruitment by dimers, as proposed for MOR^31^. This suggests that different receptors may behave differently upon dimerization, but further structural studies are required to clarify this issue.

Another interesting observation of our study is that βarrs limit oligomerization of the PAFR WT. This underlines the functional interplay between βarr and receptor quaternary structure. The influence of GPCR oligomerization on arrestin interactions has already been analyzed for GPCR heteromers, where either a decrease or increase in βarr recruitment was observed^84^. However, for the GPCR homomers, it remains largely unknown. Recently, different ligands were shown to affect the μ-opioid receptor (MOR) dimerization and βarr recruitment differently, with full agonists favoring dimers and β-arrestin interactions, while biased ligands that do not recruit arrestin not favoring MOR dimers^31^. Such data further document the possible relationship between dimerization and the control of one signaling pathway over another, although dimerization and βarr recruitment are linked in an opposite way compared to what we observed with PAFR.

Finally, our data could be of physiological or pathophysiological relevance. First, natural genetic variations in the human genome are an underappreciated public health burden. To this end, GPCR variants can differentially impact individual drug responses by potentially changing ligand potencies and efficacies, receptor conformation, surface expression, and pathway preferences^66,97^. Here, we discovered a link between natural genetic variation and PAFR dimerization which leads to signaling bias. Further studies using conditional mouse models are needed to assess the physiological or pathological effects of this natural variation. Second, high expression of PAFR was observed in the invasion and metastasis of non-small cell lung cancer and colorectal cancer^45,98^. It might lead to a larger proportion of dimers and oligomers in these cells then potentially influencing PAFR signaling and internalization. Further studies to evaluate the effects of PAFR oligomerization on tumorigenesis remain to be performed.

In conclusion, we reveal an interplay between receptor homo-oligomerization and downstream signals and provide a model for biased signaling modulation in GPCRs. According to this model, ligands that favor dimers, as reported previously^31,99^, may behave as biased agonists. However, other factors that stabilize GPCR oligomers (i.e. lipids such as cholesterol and phospholipids^100,101^) might also play a role in biased signaling. Our model provides insights into the present concept of naturally biased signaling modulation and opens new possibilities for other physiological ways to control the balance between various GPCR signaling events.

## Methods

### Materials

PAF (C26H54NO7P, 2940) was purchased from Tocris Biosciences (Ellisville, MO, USA). Acetyl-^3^H-PAF (ART0727) was purchased from American Radiolabeled Chemicals (St. Louis, MO, USA). Lipofectamine 2000 (11668019) and Fluo4-AM (F14202) were obtained from Life Technologies (Carlsbad, CA, USA). ABT-491 (A9227). Fluorescein sodium (46955), and dichloro (1,10-phenanthroline) copper(II) (CuP, 362204) were purchased from Sigma-Aldrich (St. Louis, MO, USA). Coelenterazine-h (S2011), HaloTag® Alexa Fluor®660 (G8471), and HaloTag® Alexa Fluor® 488 (G1001) were purchased from Promega Corporation (Madison, WI, USA). Halo-Lumi4-Tb (SHALOTBC) labeling reagents, βarr1 cellular kit (64BAR1TPEB), βarr2 cellular kit (64BAR2TPEB), and IP-One Gq kit (62IPAPEJ) were purchased from Perkin Elmer Cisbio (Codolet, France). Pertussis Toxin (PTX) inhibitor (HY-112779) was purchased from MedChemExpress (NJ, USA) and YM-254890 (10-1590) from Focus Biomolecules (PA, USA).

### Plasmids

The pRK5 plasmids encoding wild-type human PAFR and human β_2_AR were tagged with a double tag, either Flag-SNAP or Flag-Halo, inserted immediately after the signal peptide^57^ (Supplementary Figs. 20a, 21). Mutations in the pRK5 plasmid were generated by site-directed mutagenesis using the QuikChange mutagenesis protocol (Agilent Technologies). The probes (full-length mVenus, mCherry, or Rluc) were fused to the C terminus of PAFR or β_2_AR, respectively, with the AflII restriction site as a linker (Supplementary Figs. 20b, 22). Plasmids encoding human βarr1 and βarr2 tagged with Rluc have been described previously^97^. The plasmids encoding membrane-targeted Venus (Venus-CAAX) were obtained from Addgene^102^. The EPAC1 cDNA was obtained from Dr. Lily Jiang (University of Texas Southwestern, Dallas). cDNA for the human GRK2 and GRK3 in pcDNA3.1 was fused to Renilla luciferase (provided by ARPEGE platform, IGF).

### Cell culture and transfection

HEK-293 cells (ATCC, CRL-1573), COS-7 cells (3111C0001CCC000033, National Infrastructure of Cell Line Resources, China), and HEK-293 cells with targeted deletion of ARRB1 and ARRB2 (Δβ-arrestin1/2, were kind gifts by Dr. Asuka Inoue (Tohoku University, Sendai, Miyagi, Japan)^80^ were cultured in Dulbecco’s modified Eagle’s medium (DMEM) supplemented with 10% FBS and 100 U/ml penicillin–streptomycin at 37 °C and 5% CO_2_ in a humidified incubator. Plasmids were transfected using Lipofectamine 2000 according to the manufacturer’s recommendations^103^. On-TARGETplus Smartpool siRNAs (L-011971-00-0020) from Horizon were used to target βarr1. A nontargeting pool (D-001810-10-05) was used as a control. 60–70% confluent cells were transfected with both siRNAs (100 nM) and plasmids (100 ng/well, 96-wells plate) using Lipofectamine 2000. Data were collected in HEK-293, COS-7, or Δβ-arrestin1/2 cells, as stated in the figure legends.

### TM peptides treatment

Synthetic peptides representing each of the TM peptides for the human PAFR were obtained from the Jier (Shanghai, China) with ≥90% purity. The TM1 peptide consists of residues 15–41 (YTLFPIVYSIIFVLGVIANGYVLWVFA), TM2 peptide of residues 54–78 (IFMVNLTMADMLFLITLPLWIVYYQ), TM3 peptide of residues 88–114 (FLCNVAGCLFFINTYCSVAFLGVITYN), TM4 peptide of residues 130–156 (TRKRGISLSLVIWVAIVGAASYFLILD), TM5 peptide of residues 184–209 (VLIIHIFIVFSFFLVFLIILFCNLVI), TM6 peptide of residues 232–257 (WMVCTVLAVFIICFVPHHVVQLPWTL), and TM7 peptide of residues 270–296 (AINDAHQVTLCLLSTNCVLDPVIYCFL). Three basic sequences (KKK) were introduced at the N- and C-terminus to ensure their incorporation into the plasma membrane of cells, as demonstrated previously^55^. Before use, the peptides were solubilized in pure dimethyl sulfoxide (DMSO) and diluted in the corresponding cell culture medium to a final concentration of 10 μM. Cells were incubated with the peptides mentioned above at 37 °C for 2 h before performing BRET analysis.

### Cell surface receptor quantification by ELISA

Flag-tagged subunits were transfected into the indicated cells and seeded in white transparent 96-well plates. 24 h after transfection, the HEK-293 cells were fixed in 4% paraformaldehyde. Cell surface expression was detected with a monoclonal mouse anti-Flag antibody M2 at 0.8 mg/ml (F1804, Sigma-Aldrich) and a goat anti-mouse secondary antibody coupled to horseradish peroxidase at 0.25 mg/ml (115-035-003, Jackson Immunoresearch, West Grove, PA, USA)^103^. The bound antibody was detected by chemiluminescence using the SuperSignal substrate (Thermo Fisher Scientific) and Flexstation 3 (Molecular Devices, Sunnyvale, CA, USA).

### Radioactivity binding assay

24 h after transfection, cells were washed one time with binding buffer (PBS containing 0.2% w/v free fatty acid bovine serum albumin, 1.3 mM CaCl_2_, 1 mM MgCl_2_ at pH 7.4). [^3^H]-PAF concentration in the range from 0.1 to 500 nM was used. Specific binding was determined as the total radioactivity bound minus the radioactivity bound in the presence of 10 mM unlabeled PAF as non-specific binding. The reaction mixture was incubated on ice for 2 h and then washed three times with binding buffer. Afterward, cells were lysed with 0.1 M NaOH and counted for radioactivity in a scintillation counter (Packard Instrument Co.). The total cell protein concentration was determined by the method of the bicinchoninic acid assay using bovine serum albumin as a standard. The maximal concentrations of binding sites (*B*_max_) were determined by Scatchard plots. Results were expressed as pmol [^3^H]-PAF/mg of total cell proteins^53^.

### Halo labeling and total internal reflection fluorescence (TIRF) microscopy

COS-7 cells were transfected with the indicated constructs using Lipofectamine 2000 and plated in a 35 mm confocal dish. 24 h after transfection, cells were labeled with HaloTag® Alexa Fluor® 488 (1 μM) for 30 min at 37 °C and fixed with 4% paraformaldehyde for 15 min. Then, the cells were washed thrice with PBS, and images were taken.

A TIRF microscope, equipped with a high-NA TIRF objective (Olympus Oil ×100 or ×150, NA = 1.45) and an electron-multiplying charge-coupled device (AndoriXon DV-897 BV), was adopted to achieve single-molecule detection, and a solid-state 488-nm laser (OPSL, Coherent) with 1 mW was used. To avoid photobleaching before image acquisition, cells were searched and focused in a bright field, and a fine focus adjustment in TIRF mode was performed using only 2% laser power. This procedure results in negligible photobleaching. Afterward, the laser power was set to 83%, and image sequences (1000 frames) were acquired with an exposure time of 50 s, resulting in the acquisition of an image every 50 ms. Sequences of images (records) were stored directly on a computer hard drive for subsequent analysis.

Imaging data were analyzed by a home-written MATLAB (Version 1.0.0.1, MathWorks) code^104^. Briefly, an “à trous” wavelet filter was applied to each frame to extract all the single molecules. Each isolated molecule was fitted to a 2D Gaussian function to obtain the precise *x*–*y* location, as well as the intensity and background. To find the corresponding single molecule in successive frames, a well-established trajectory linking algorithm (http://site.physics. georgetown.edu/matlab/) was adopted. For each identified trace, the bleaching steps were determined manually by one investigator and rescored blindly by another.

### Photobleaching step analysis

Since the labeling efficiency of the fluorescent probe is far from 100%, the mono/di/oligomeric fraction calculated from the photobleaching step required further correction. The binding between dye and protein units was treated as an independent random event for statistical analysis. The probability of a single subunit binding is ${P}_{{\rm {f}}}$ which is measured by actual experiments. The probability that an *N*-order oligomer to be observed as an *m*-order is thus

$${P}_{{Nm}}=\left({m}\atop{N}\right){{P}_{f}}^{m}{(1-{P}_{f})}^{N-m}$$

Hence, the ratio of *m*th-order oligomer ${R}_{m}$ observed in the photobleaching experiment can be expressed as

$${R}_{m}=\mathop{\sum }\limits_{i=m}^{N}\left({m}\atop{i}\right){{P}_{f}}^{m}{(1-{P}_{f})}^{i-m}$$

A system of linear equations ${A}_{M\times N}$ is constructed according to the probability model and the corrected oligomer fraction ${C}_{m}$ is the solution of ${A}_{M\times N}{x}_{N\times 1}={R}_{M\times 1}$ . As the high-order polymer has little effect on our experiment data, our correction only takes monomer/dimer /trimer/tetramer/pentamer into account.

### Cross-linking and fluorescent-labeled blot experiments

24 h after transfection, adherent HEK-293 cells plated in 12-well plates were labeled with 300 nM Halo-660 in culture medium at 37 °C for 2 h. Then, cells were incubated with the drug (each at 10 μM) or PBS at 37 °C for 15 min. Afterwards cross-link buffer (1.5 mM Cu(II)-(*o*-phenanthroline), 1 mM CaCl_2_, 5 mM MgCl_2_, 16.7 mM Tris–HCl, pH 8.0, 100 mM NaCl) was added at 20 °C for 30 min. After incubation with 10 mM N-ethylmaleimide at 4 °C for 15 min to stop the cross-linking reaction, cells were lysed with lysis buffer (50 mM Tris–HCl, pH 7.4, 150 mM NaCl, 1% Nonidet P-40, 0.5% sodium deoxycholate, 0.1% SDS, and protease inhibitors) at 4 °C for 1.5 h. After centrifugation at 12,000 × *g* for 30 min, supernatants were warmed up with loading buffer (NuPAGE LDS sample buffer 4, Invitrogen) at 37 °C for 10 min. Electrophoresis was performed and the proteins were blotted onto nitrocellulose membranes. Membranes were imaged using an Odyssey infrared scanner (LI-COR Biosciences, Lincoln, NE, USA) at 700 nm for Halo-Red^57^.

### Diffusion-enhanced resonance energy transfer internalization assay

A diffusion-enhanced resonance energy transfer (DERET) internalization assay was performed for measuring PAFR internalization in real time^63^. Transfected HEK-293 cells in black non-transparent 96-well plates were labeled in Tag-lite labeling buffer with 100 nM Halo-Lumi4-Tb for 1.5 h at 4 °C. Excess of Lumi4-Tb was removed by washing each well four times with 100 μl of Tag-lite labeling buffer. Internalization experiments were performed by incubating the cells with a Tag-lite-labeling medium, either alone or containing PAF, in the presence of fluorescein. Typically, in plates containing Lumi4-Tb-labeled cells, 50 μl of buffer containing PAF at the indicated concentrations was added, immediately followed by the addition of 50 μl of 48 μM fluorescein. Afterward, the cells were incubated at 37 °C for 1 h.

Lumi4-Tb was excited by a laser at 337 nm, and the emission fluorescence intensities were recorded for the donor (620 nm, 1500 μs delay, 1500 μs reading time) and acceptor (520 nm, 150 μs delay, 400 μs reading time) using a PHERAstar FS microplate reader (BMG Labtech, Ortenberg, Germany). The ratio of 620/520 was obtained by dividing the donor signal (620 nm) by the acceptor signal (520 nm) and multiplying this value by 10,000. Data are expressed as the percentage of maximal internalization and were normalized as indicated.

### Fluorescence labeling for quantification and oligomerization of PAFR

For cell surface quantification, SNAP-tagged PAFR was labeled with 100 nM SNAP-Lumi4-Tb at 4 °C for 1 h. After being labeled, cells were washed thrice with Tag-lite buffer. For measurement of PAFR oligomerization at the cell surface, SNAP-tagged PAFR were labeled, 24 or 48 h after transfection, by incubation at 4 °C for 1 h with a solution of 100 nM SNAP-Lumi4-Tb and 300 nM SNAP-green in Tag-lite buffer (Perkin-Elmer Cisbio). TR-FRET measurements were performed using a PHERAstar FS microplate reader. After excitation with a laser at 337 nm (40 flashes per well), the fluorescence was collected at 520 nm for a 50 μs reading after a 50 μs delay after excitation (window 1) or for a 400 μs reading after a 1200-μs delay (window 2). The acceptor ratio was determined by dividing the signal measured in window 1 by the signal measured in window 2^105^.

### Intracellular calcium release

Briefly, a plasmid of interest was transfected into the indicated cells seeded into black transparent 96-well microplates for at least 24 h. The cells were then preincubated for 1 h with Ca^2+^-sensitive Fluo-4 (Life Technologies). The fluorescence signals (excitation at 485 nm and emission at 525 nm) were measured for 60 s (Flexstation 3, Molecular Devices) and recorded using a Flexstation 3 microplate reader or FLIPR Tetra (Molecular Devices, Sunnyvale, CA, USA). The agonist was added after the first 20 s. The Ca^2+^ response is expressed as an agonist-stimulated increase in fluorescence^103^.

### IP_1_ measurements

IP_1_ accumulation in HEK-293 cells was measured using the IP One HTRF kit (Perkin Elmer Cisbio), according to the manufacturer’s recommendations.

### BRET assays

For the BRET measurements between protomers, increasing amounts of Venus-tagged receptors were co-expressed with constant amounts of Rluc-tagged receptors in HEK293 cells^106^ (Supplementary Fig. 20b). 24 h after transfection, the coelenterazine *h* substrate was added at a final concentration of 5 μM. BRET signals (emission light at 480 and 530 nm, respectively) were detected by Mithras LB940 (Berthold Technologies GmbH & Co., KG). The BRET signals were plotted against the relative expression levels of each tagged receptor. netBRET ratio = [YFP emission at 530/Rluc emission 480] (where PAFR-Rluc or β_2_AR-Rluc are cotransfected with PAFR-Venus)−[YFP emission at 530/Rluc emission 480] (where PAFR-Rluc or β_2_AR-Rluc are transfected alone), in the same experiment. The results were analyzed by nonlinear regression assuming a model with one-site binding (GraphPad Prism, version 9, GraphPad software) on a pooled dataset from three independent experiments.

For β-arrestin recruitment assay using ebBRET assay, βarr1-Rluc or βarr2-Rluc (BRET donor), Venus-CAAX (BRET acceptor), and PAFR were co-transfected into cells for at least 24 h. For BRET assay between βarrs and the receptor, Venus-tagged PAFR and βarr1-Rluc or βarr2-Rluc were co-transfected into cells for at least 24 h. Before detection, cells were starved with PBS at 37 °C for 30 min. Afterward, the coelenterazine *h* substrate was added at a final concentration of 5 μM, and BRET signals were detected by Mithras LB940 (Berthold Technologies) with stimulation by PBS or an agonist.

For GRK2 and GRK3 recruitment using BRET assay^107^, Venus-tagged PAFR and GRK2-Rluc or GRK3-Rluc were co-transfected into cells for at least 24 h. Before detection, cells were starved with PBS at 37 °C for 30 min. Afterward, the coelenterazine *h* substrate was added at a final concentration of 5 μM, and BRET signals were detected by Mithras LB940 with stimulation by PBS or an agonist.

For the cAMP measurement by EPAC BRET sensor^108^, PAFR and EPAC1 were co-transfected into cells for at least 24 h. Before detection, cells were starved with PBS at 37 °C for 30 min. Afterward, the coelenterazine *h* substrate was added at a final concentration of 5 μM, and BRET signals were detected by Mithras LB940 with stimulation by forskolin (10 μM) to produce cAMP, and further stimulation by PAF to inhibit cAMP production.

### β-arrestin-1 and β-arrestin-2 expressions

βarr1 and βarr2 expressions in cells were measured using a total βarr1 and βarr2 cellular kit (Perkin Elmer Cisbio), according to the manufacturer’s recommendations. TR-FRET measurements were performed using a PHERAstar FS microplate reader. After excitation with a laser at 337 nm (40 flashes per well), the fluorescence was collected at 620 and 665 nm for a 400 μs reading after a 60 μs delay after excitation. The ratio between 665 and 620 nm was determined.

### Molecular modeling

The PAFR 7TM structure was retrieved from the PDB 5ZKQ. Residues to be substituted with cysteine were retrieved from GPCRdb (www.gpcrdb.org), and substitutions were highlighted using PyMOL (Version 2.5, Schrödinger). Natural genetic variants of PAFR were also obtained from GPCRdb. The structures of the complexes between GPCR and arrestin were retrieved from the PDB 6U1N for muscarinic M_2_ receptor and βarr1 complex, PDB 6TKO for β_1_-adrenergic receptor and βarr1 complex, 5W0P for rhodopsin and arrestin-1 complex, 6UP7 for neurotensin NTS1 receptor and βarr1 complex), and analyzed using PyMOL.

### Curve fitting and data analysis

All data in the figures and the supplementary figures are mean ± SEM of at least three independent experiments performed in triplicate unless stated differently in the figure legends. The curves were fitted using Prism software (Version 9, GraphPad Software). Fluorescent images were analyzed using lmageJ (Version 1.440, National Institutes of Health). Single molecular imaging data were analyzed using MATLAB (Version 1.0.0.1, MathWorks). Statistical differences were calculated using GraphPad Prism. *P*-values were determined using unpaired two-tailed *t*-test, paired two-tailed *t*-test, or one-way ANOVA with Dunnett’s multiple comparisons test. Differences were considered to be not significantly different (ns) when *P*  > 0.05.

### Reporting summary

Further information on research design is available in the Nature Research Reporting Summary linked to this article.

## Supplementary information

Supplementary Information

Reporting Summary

## Data Availability

Data supporting the findings of this manuscript are available from the corresponding authors upon request. Source data are provided with this paper.

## Source data

Source Data
