# Supplementary material for: Biased signaling due to oligomerization of the G protein-coupled platelet-activating factor receptor
Source: Nat Commun. 2022 Oct 26;13:6365. doi: 10.1038/s41467-022-34056-4 (PMC9606269; doi:10.1038/s41467-022-34056-4)
Supplement: Supplementary file 1 — Supplementary Information [file 41467_2022_34056_MOESM1_ESM.pdf]

Biased signaling due to oligomerization of the G protein-coupled platelet-activating factor receptor

Junke Liu[1,2,5], Hengmin Tang[1,5], Chanjuan Xu[1], Shengnan Zhou[1], Xunying Zhu[1], Yuanyuan Li[3], Laurent Prézeau[2], Tao Xu[3,4], Jean-Philippe Pin[2*], Philippe Rondard[2*], Wei Ji[3,4*], Jianfeng Liu[1,4*]

[1]Cellular Signaling laboratory, International Research Center for Sensory Biology and Technology of MOST, Key Laboratory of Molecular Biophysics of MOE, College of Life Science and Technology, Huazhong University of Science and Technology, 430074 Wuhan, Hubei, China;

[2]Institut de Génomique Fonctionnelle, Université de Montpellier, CNRS, INSERM, 34094 Montpellier Cedex 5, France;

[3]National Laboratory of Biomacromolecules, CAS Center for Excellence in Biomacromolecules, Institute of Biophysics, Chinese Academy of Sciences, Beijing, China;

[4]Guangzhou Regenerative Medicine and Health Guangdong Laboratory, Chinese Academy of Sciences, 510005 Guangzhou, China;

[5]These authors contributed equally.

*Correspondence to:

jfliu@mail.hust.edu.cn;

jiwei@ibp.ac.cn;

philippe.rondard@igf.cnrs.fr

jean-philippe.pin@igf.cnrs.fr

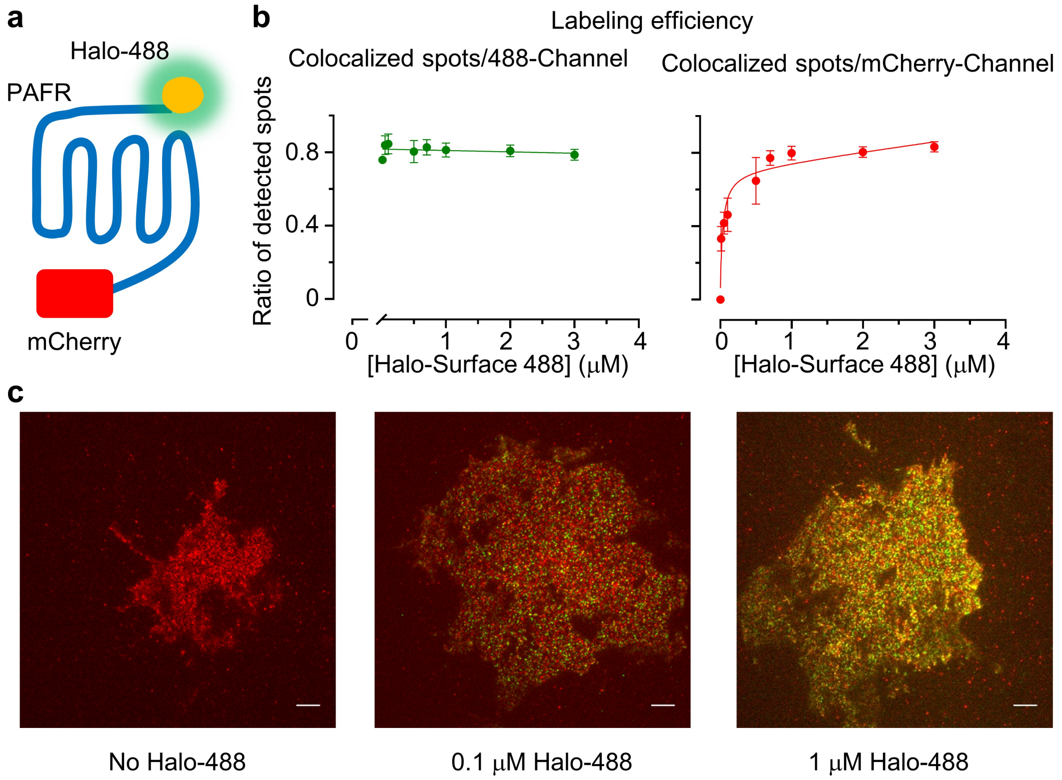

Supplementary Figure 1. Labeling efficiency and optimal labeling concentration. (a) Schematic representation of the control construct Halo-PAFR N-terminally labeled with non-cell permeant Halo-Surface 488 (Halo-488) and C-terminally tagged with mCherry (see Supplementary Figure 20b for the details of the construct). **(b)** Plots show ratios based on the amounts of detected molecules in each detection channel against the dye concentration used. Data are mean ± SEM from n = 6 different cells for each concentration. **(c)** TIRF images of the control construct where green particles correspond to Halo-Surface 488 and the red ones to mCherry tagged receptors. Successfully tagged constructs appear as yellow spots, while red spots represent the receptors unlabeled with the dye and green spots represent the unspecific binding to other cell-surface structures. Data are representative of a typical experiment from n=6 different cells for each condition. Scale bar = 5 μm.

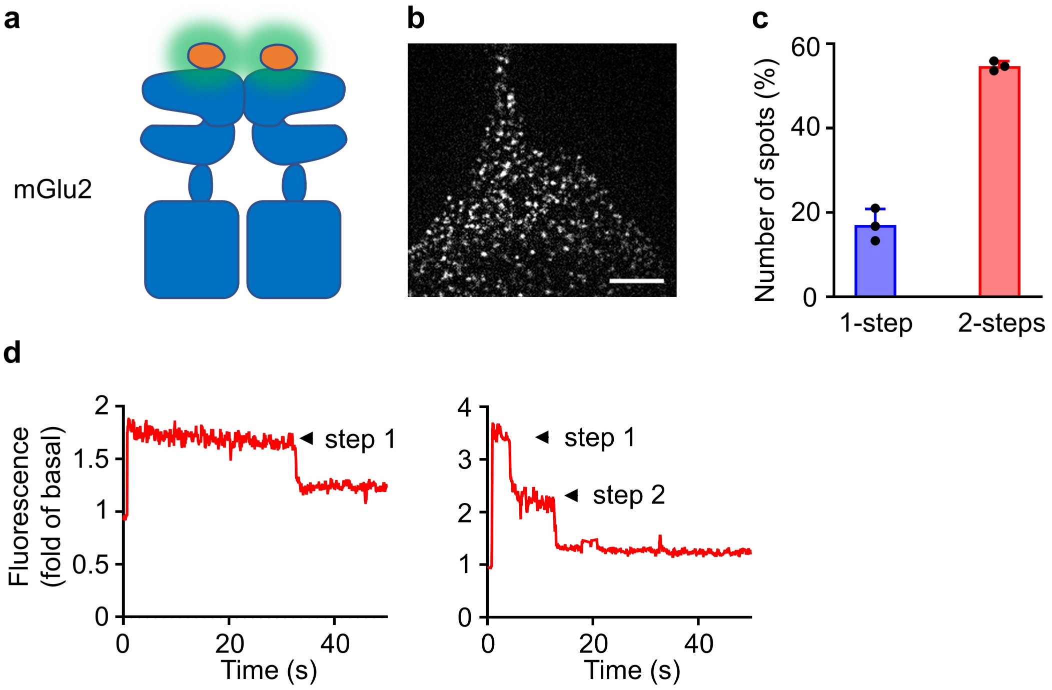

Supplementary Figure 2. Photobleaching step analysis of the homodimeric mGlu2 receptor. (a) Schematic representation of the obligatory Halo-tagged mGlu2 dimer and labeling with non-cell permeant Halo-488 for the TIRF imaging. **(b)** TIRF image of Halo-mGlu2 subunits. Data are representative of a typical experiment from n=3 biologically independent experiments. Scale bar = 5 μm. **(c)** Photobleaching step analysis for the Halo-mGlu2 subunits. Analyzed spots are from more than seven movies for one experiment and repeated three times (2382 spots from 23 movies). Data are mean ± SEM from n=3 biologically independent experiments. **(d)** Representative fluorescence time course for an individual molecule for the one-step (left) and two-steps (right) photobleaching.

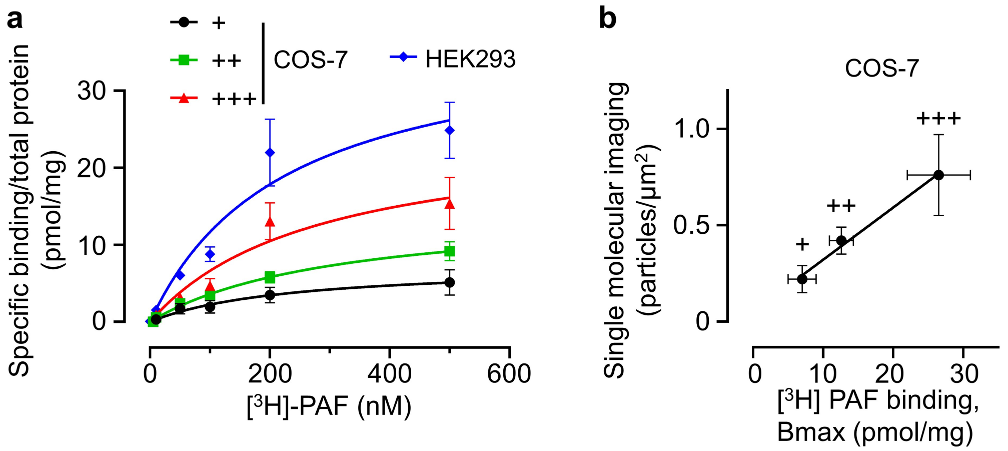

Supplementary Figure 3. Binding of [^{3}H]-PAF to COS-7 and HEK-293 cells expressing different densities of the human PAF receptor. **(a)** Specific binding was calculated as (total binding – non-specific binding). Data are mean ± SEM from n=4 biologically independent experiments. We transfected similar amount receptors for COS-7 cells in Figure 1 (+, ++, +++) and for HEK-293 cells in the different assays (Ca^{2+} release and IP_1 assays and BRET assays for βarr recruitment). The B_{max} values were determined by Scatchard transformations of binding data. In COS-7 cells, the B_{max} were +, 7.0 ± 2.0 pmol/mg of total cell proteins; ++, 12.6 ±1.7 pmol/mg; +++, 26.5 ± 4.5 pmol/mg, and in HEK-293 cells, 41.4 ± 7.2 pmol/mg of total cell proteins. **(b)** Correlation between the receptor density measured by single particle counting, and the B_{max} measured by radioactivity ligand binding experiments. Data are mean ± SEM from n=3-4 biologically independent experiments.

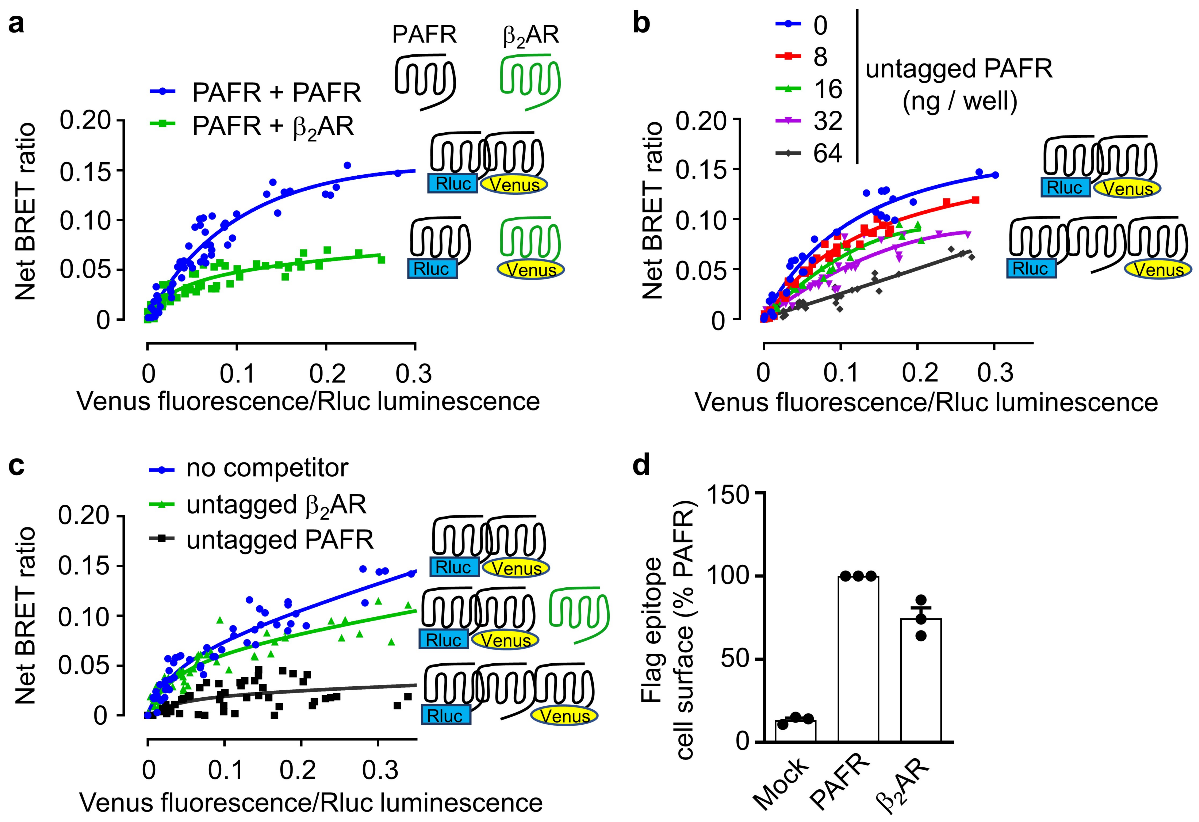

Supplementary Figure 4. **Analysis of PAFR oligomerization by BRET assay. (a)** BRET titration curves were obtained in HEK-293 cells cotransfected with a constant amount of Rluc-tagged PAFR or β_2AR cDNA and increasing amounts of Flag-tagged PAFR-Venus cDNA. **(b-c)** BRET titration curves were obtained in HEK-293 cells cotransfected with a constant amount of Rluc-tagged PAFR and increasing amounts of Flag-tagged PAFR-Venus, in the absence or presence of different amounts of untagged PAFR *(b)*, or in the absence or presence of untagged PAFR or β_2AR as indicated *(c)*. Data in *a-c*, are pooled data set from n=3 biologically independent experiments and were analyzed by nonlinear regression assuming a model with one-binding site. Of note, the data in presence of β_2AR are significantly below the condition in absence of competitor. **(d)** ELISA measurement of the amount of Flag-tagged indicated receptors at the cell surface of transfected HEK-293 cells. Data are mean ± SEM from n=3 biologically independent experiments.

a

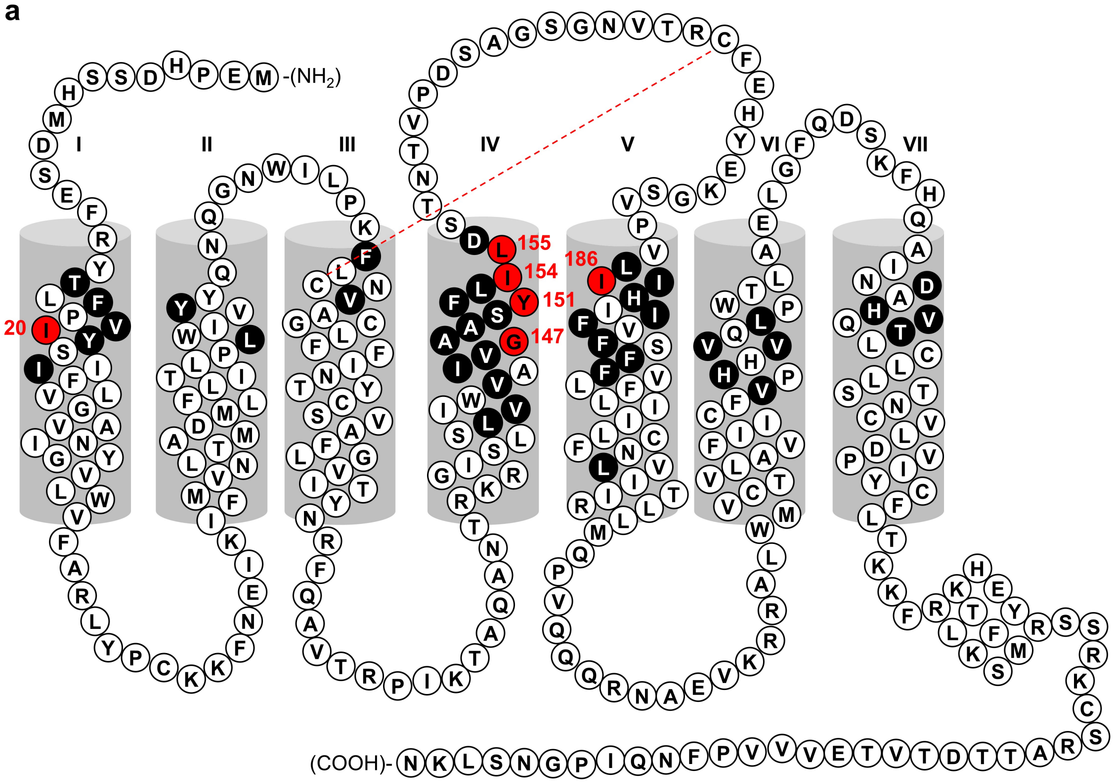

b

Cys mutations													
TM1		TM2		TM3		TM4		TM5		TM6		TM7	
1.33	T16C	2.59	L72C	3.23	F88C	4.47	L139C	5.37	L185C	6.49	V246C	7.33	D273C
1.35	F18C	2.63	Y76C	3.27	V92C	4.48	V140C	5.38	I186C	6.51	H248C	7.35	H275C
1.37	I20C					4.51	V143C	5.39	I187C	6.53	V250C	7.37	V277C
1.38	V21C					4.53	I145C	5.40	H188C	6.54	V251C	7.38	T278C
1.39	Y22C					4.54	V146C	5.42	F190C	6.56	L253C		
1.41	I24C					4.55	G147C	5.43	I191C				
						4.56	A148C	5.45	F193C				
						4.57	A149C	5.47	F195C				
						4.58	S150C	5.48	F196C				
						4.59	Y151C	5.59	L207C				
						4.60	F152C						
						4.61	L153C						
						4.62	I154C						
						4.63	L155C						
						4.64	D156C						

Supplementary Figure 5. (a) Secondary structure of the 7TM domain of the human WT PAFR. Amino acids that were replaced with cysteine residues are highlighted by black circles. The residues that can be cross-linked in each TM domain are highlighted in red. **(b)** PAFR mutants in which the indicated single mutation was introduced in different TM domains. The positions where the introduction of a Cys in PAFR led to efficient cross-linking between protomers are highlighted in red.

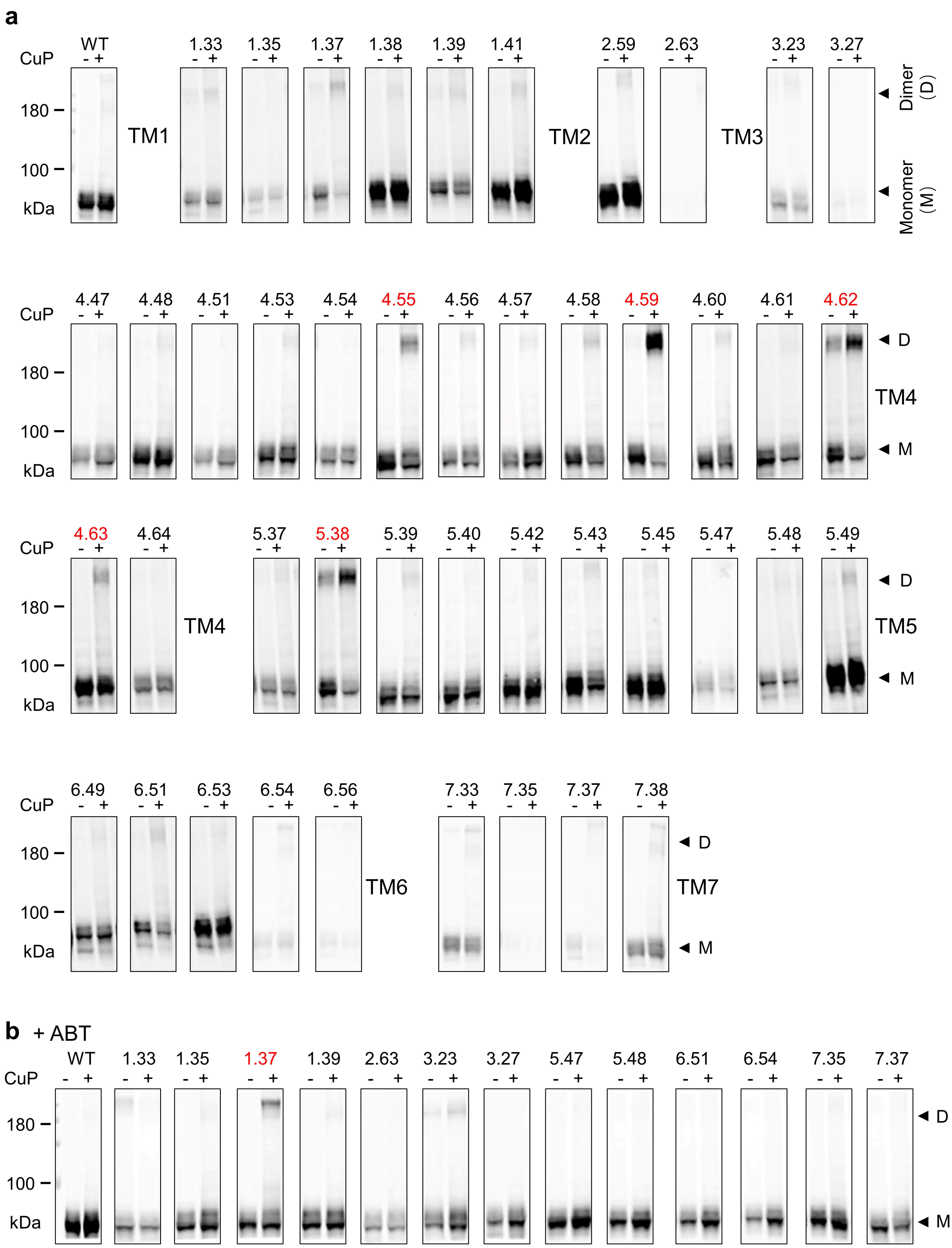

Supplementary Figure 6. Cross-linking of cell surface Halo-PAFR. (a-b) Cross-linking of the indicated cell surface Halo-PAFR protomers labeled with fluorescent Halo substrates, after treatment (+) or without treatment (−) with CuP. After SDS-PAGE in nonreducing conditions, Halo-PAFR monomers and dimers were detected via the fluorophore covalently attached to the

receptors. ABT-491 (1 μM) was used to improve the expression of indicated mutants (*b*). Of note, the constructs highlighted in red are those that produced a large increase in the ratio between PAFR dimer over the total of subunit after CuP treatment (see Figure 2c, 2d). Data are representative of a typical experiment from n=3-7 biologically independent experiments.

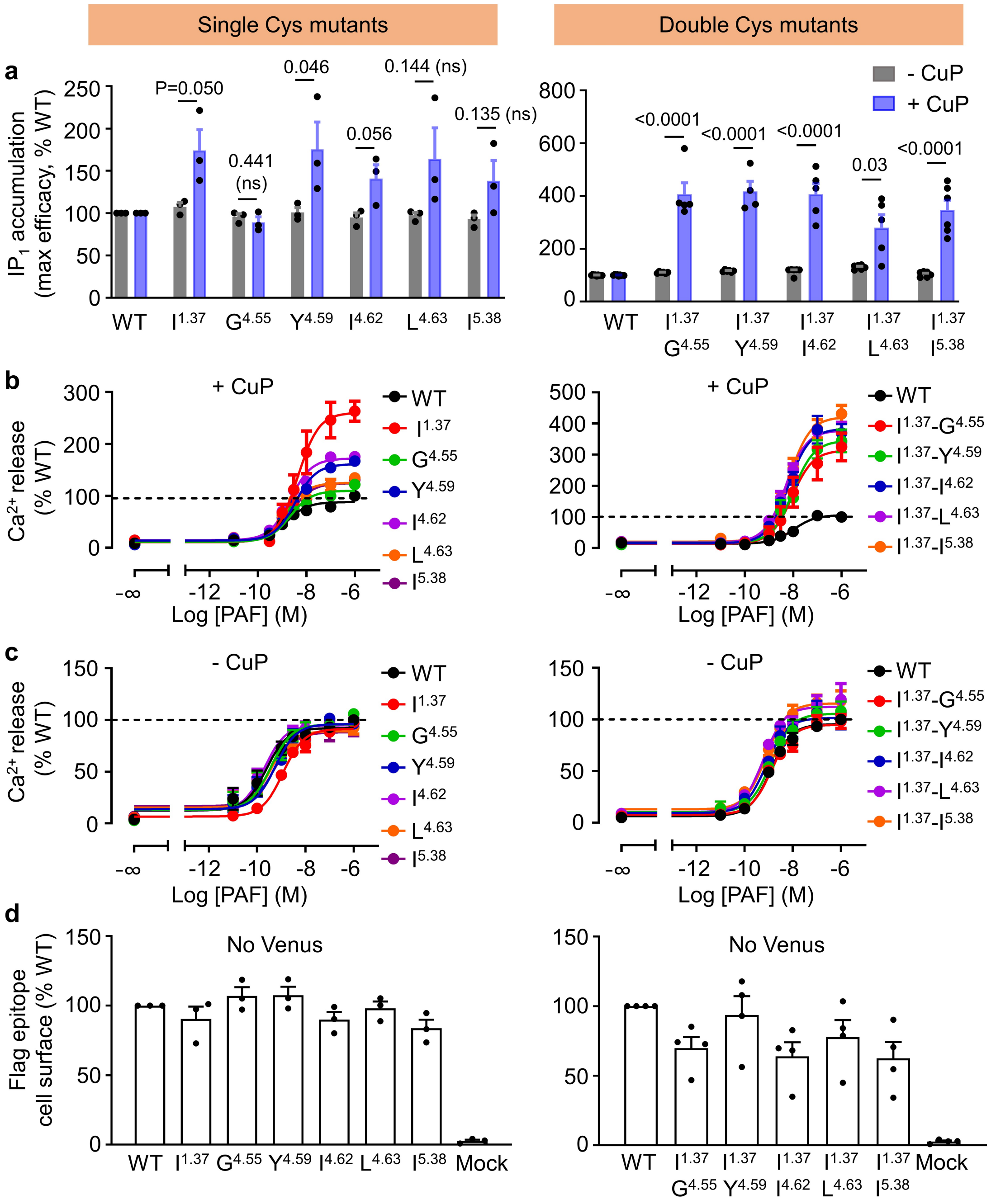

Supplementary Figure 7. Oligomerization of PAFR increases Gq signaling. (a) IP_1 accumulation mediated by the indicated Flag-PAFR mutants upon stimulation with PAF (1 μM), after or without treatment with CuP. Data are mean ± SEM from n=3-6 biologically independent experiments and normalized to WT, analyzed using one-way ANOVA with Dunnett's multiple comparisons test. **(b, c)** Intracellular Ca^{2+} responses mediated by the indicated cysteine substitutions upon stimulation with PAF, after or without treatment with CuP. Data are mean ± SEM from n=3 biologically independent experiments performed in triplicates and normalized to WT. **(d)** ELISA measurement of the amount of the indicated Flag-tagged PAFR (without Venus) at the cell surface of transfected HEK-293 cells. Data are mean ± SEM from n=3 biologically independent experiments.

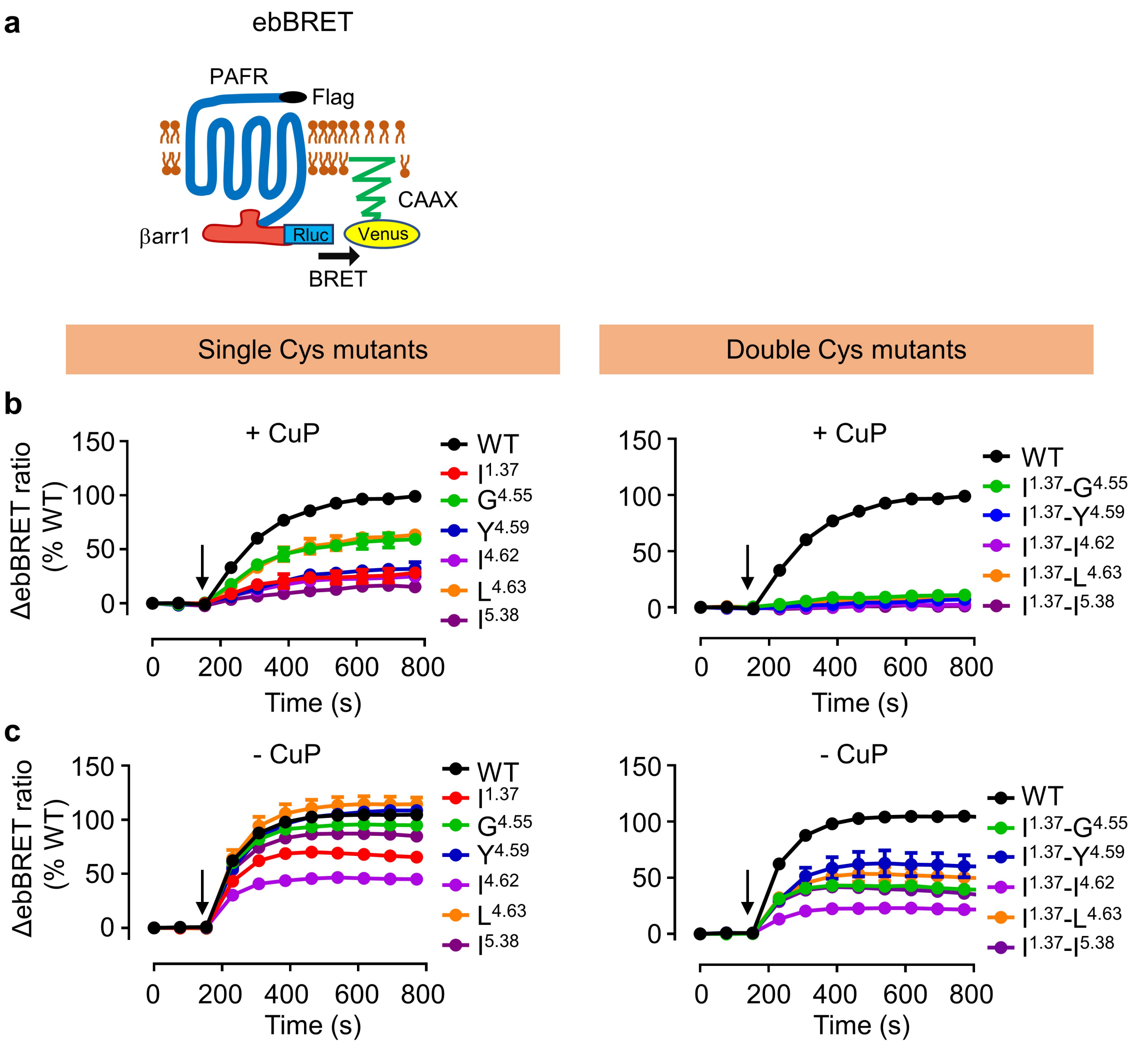

Supplementary Figure 8. Oligomerization of PAFR prevents β-arrestin recruitment to the plasma membrane. (a) Scheme illustrating the bystander BRET assay to measure the recruitment of βarr1-Rluc to the plasma membrane labelled with Venus-CAAX upon agonist activation of PAFR in HEK-293 cells. **(b-c)** Kinetics of the BRET signal after injection of PAF (1 µM, arrow) for the indicated Flag-tagged PAFR mutants, after or without treatment with CuP. Data are mean ± SEM from n=3 biologically independent experiments performed in triplicates and normalized to WT.

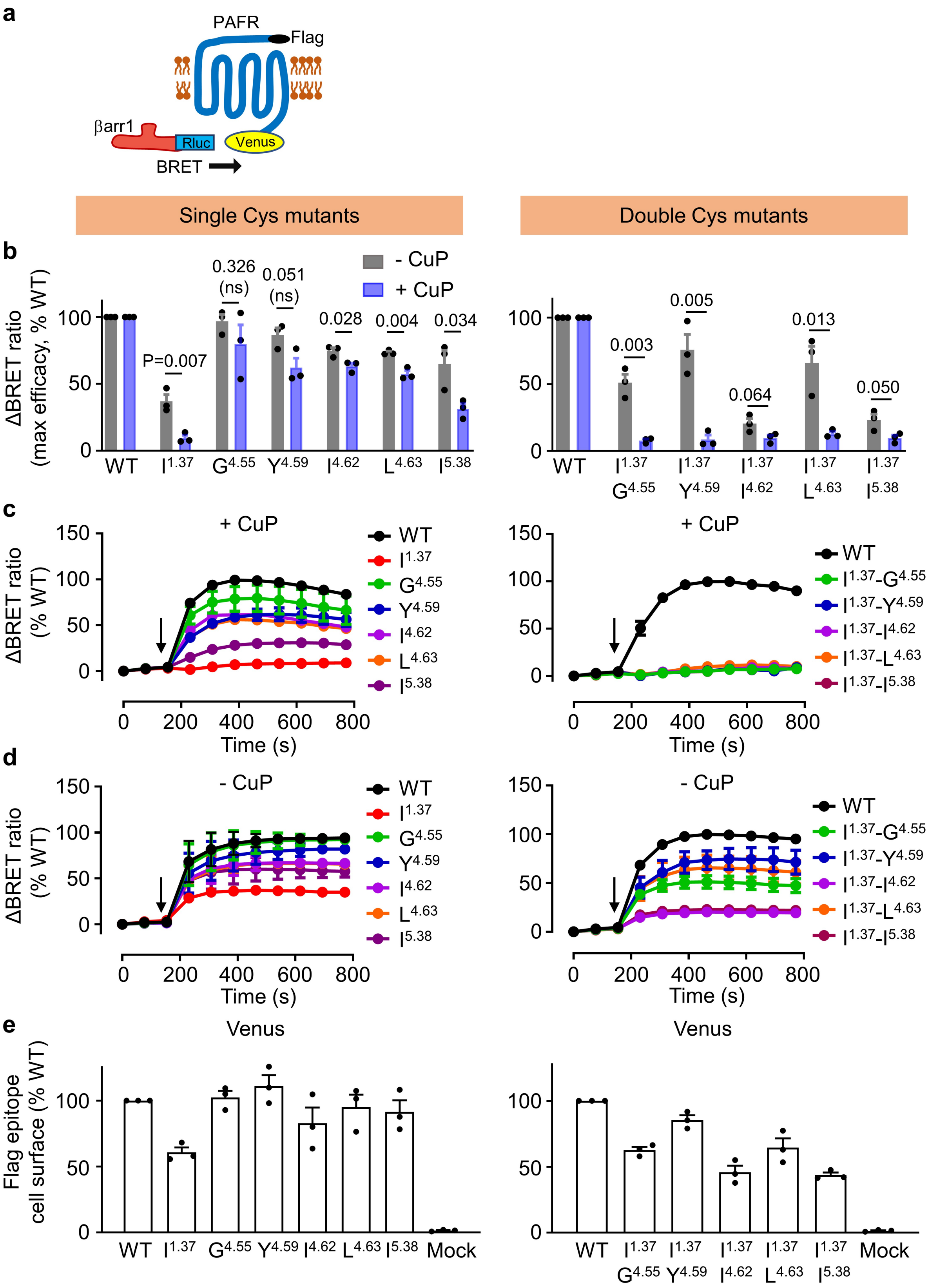

Supplementary Figure 9. Oligomerization of PAFR prevents β-arrestin recruitment to the receptor. (a) Scheme illustrating the BRET assay to measure the recruitment of βarr1-Rluc to Flag-tagged PAFR-Venus in HEK-293 cells. **(b-d)** BRET signal of βarr1-Rluc recruitment

to the indicated Flag-tagged PAFR-Venus after injection of PAF (1 μM, arrow), after or without treatment with CuP. Maximal efficacy *(b)* of the BRET signal is from panels *c* and *d*. Data are mean ± SEM from n=3 biologically independent experiments performed in triplicates and normalized to WT, and analyzed using one-way ANOVA with Dunnett's multiple comparisons test. **(e)** ELISA measurement of the amount of the indicated Flag-tagged PAFR-Venus at the cell surface of transfected HEK-293 cells. Data are mean ± SEM from n=3 biologically independent experiments performed in triplicates and normalized to WT.

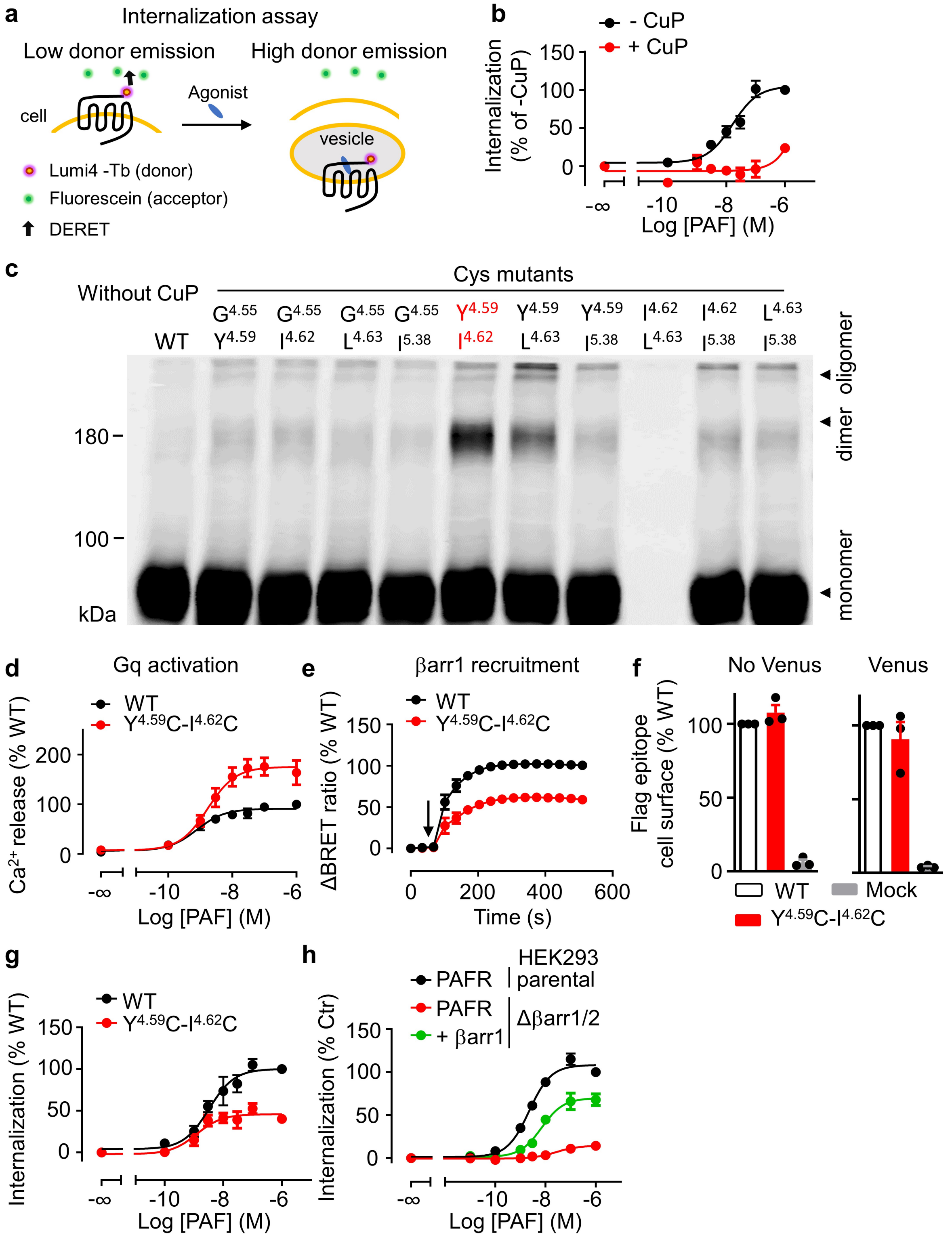

Supplementary Figure 10. PAFR oligomers distinctly modulate G protein activation, β-arrestin recruitment and receptor internalization. (a) Diffusion-enhanced resonance energy transfer internalization assay. SNAP or Halo-tagged receptors at the cell surface are labeled covalently with non-cell permeable SNAP or Halo-Lumi4®-Tb (energy donor, purple). When

the receptor is at the cell surface, addition of a free energy acceptor (fluorescein, green) to the cell medium leads to efficient energy transfer, quenching the donor luminescence and resulting in a low donor emission. Following agonist-induced internalization of the receptor, energy transfer to the acceptor is reduced, increasing the donor emission. **(b)** Agonist-induced internalization of WT PAFR after or without treatment with CuP. Data are mean ± SEM from n=3 biologically independent experiments performed in triplicates and normalized to without treatment of CuP. **(c)** Cross-linking of the indicated subunits containing Cys substitutions in both TM4 and TM5 without treatment with CuP. Data are representative of a typical experiment from n=3 biologically independent experiments. **(d)** Intracellular Ca^{2+} responses mediated by the indicated Flag-tagged PAFR mutant in absence of CuP treatment and comparison to the WT PAFR. **(e)** Kinetics of the BRET signal between the indicated Flag-tagged PAFR-Venus mutant and βarr1-Rluc after injection of PAF (1 μM, arrow) in absence of CuP treatment and comparison to the WT PAFR. **(f)** ELISA measurement of the amount of the indicated Flag-tagged PAFR (with or without Venus) at the cell surface of transfected HEK-293 cells. **(g)** Agonist-induced internalization of the indicated double mutant and WT PAFR. **(h)** Agonist-induced internalization of the WT PAFR upon stimulation with PAF in parental (Ctr) and Δβarr1/2 HEK-293 cells. In *d-h*, data are mean ± SEM from n=3 biologically independent experiments performed in triplicates and normalized to WT or Ctr.

a

Natural genetic variant (NGV)						
TM1		TM4		TM5		ECL2
1.38	V21I	4.52	A144S	5.45	F193S	E178K
1.41	I24T	4.62	I154T			
1.45	L28F	4.63	L155P			

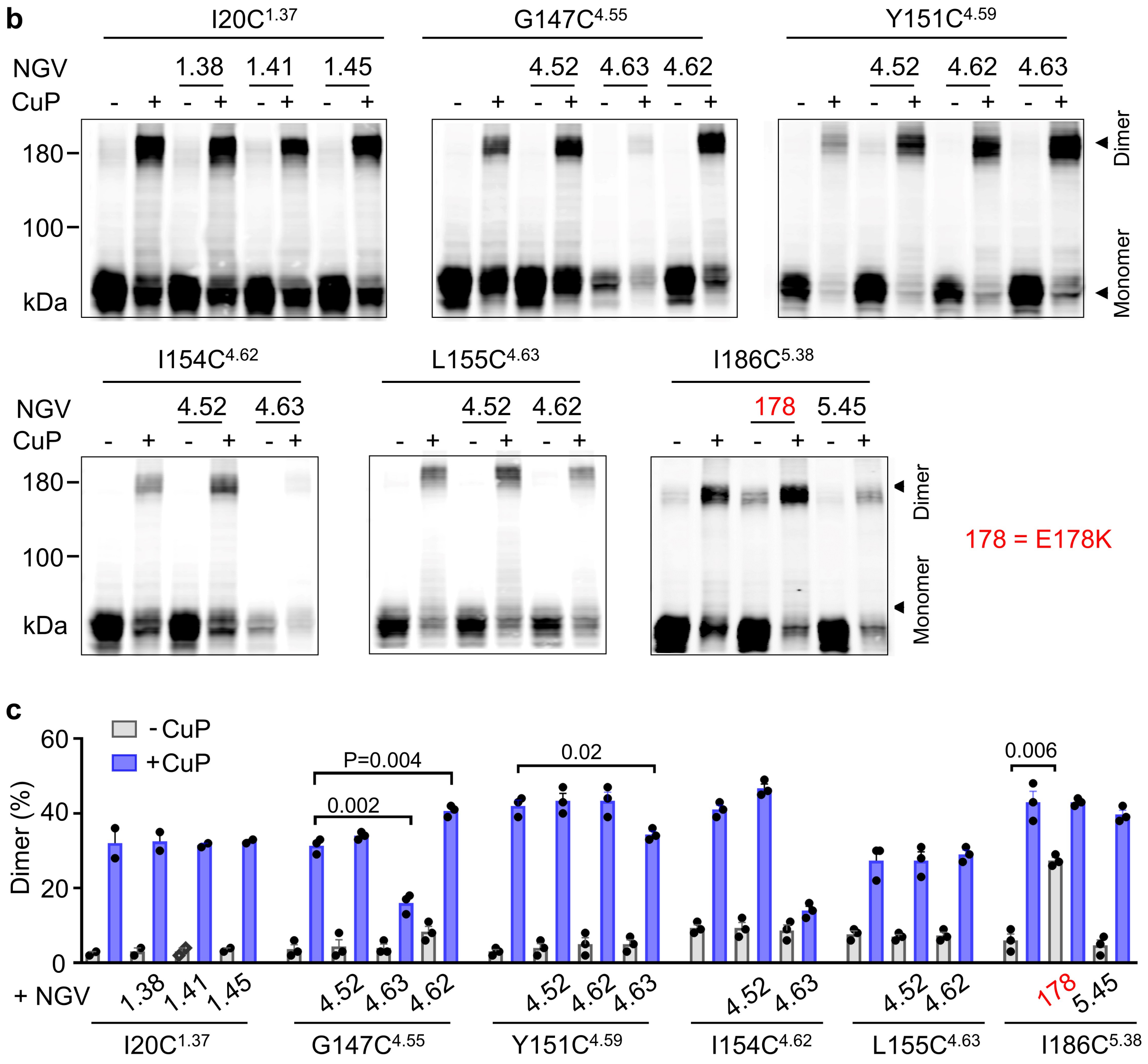

Supplementary Figure 11. Effect of natural genetic variants on PAFR dimerization. (a) Natural genetic variants investigated in this study, and the one with the most interesting effect is highlighted in red, including in *b*. **(b)** Blots showing cross-linking of cell surface Halo-PAFR subunits containing a cysteine substitution in TM1, TM4 or TM5 as indicated, and the indicated variant, with or without treatment with CuP. Data are representative of a typical experiment from n=3 biologically independent experiments. **(c)** Quantification of the PAFR dimers from the blots in *b* with or without treatment with CuP. Data are mean ± SEM from n=3 biologically independent experiments and analyzed using paired two-tailed *t*-test.

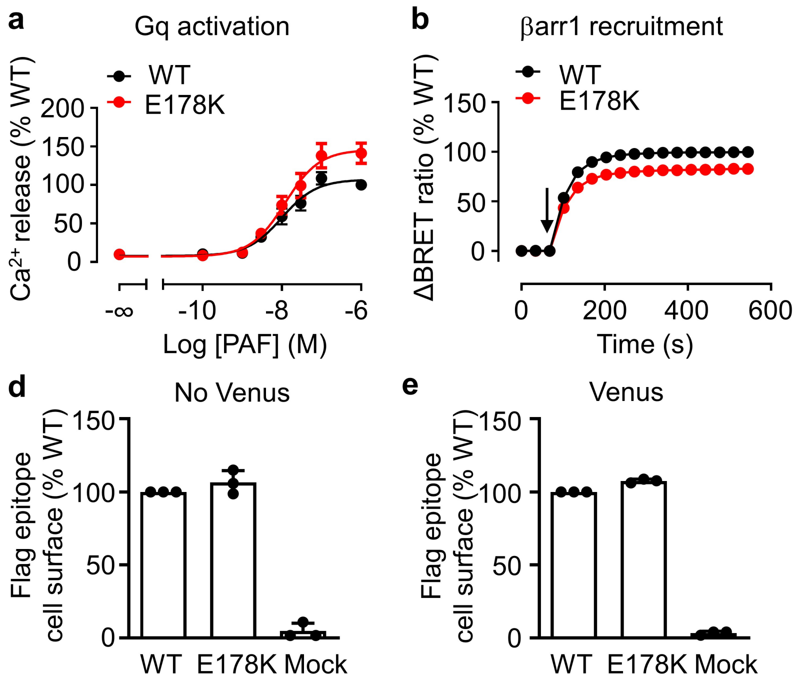

Supplementary Figure 12. Expression and functional analysis of the E178K genetic variant. (a) Intracellular Ca^{2+} responses. **(b)** βarr1 recruitment upon stimulation with PAF (1 μM, arrow). **(c)** Agonist-induced internalization. **(d-e)** Cell surface expression by ELISA assay of the Venus-tagged or none Venus-tagged PAFR. In *a-e*, data are mean ± SEM from n=3 biologically independent experiments performed in triplicates and normalized to WT.

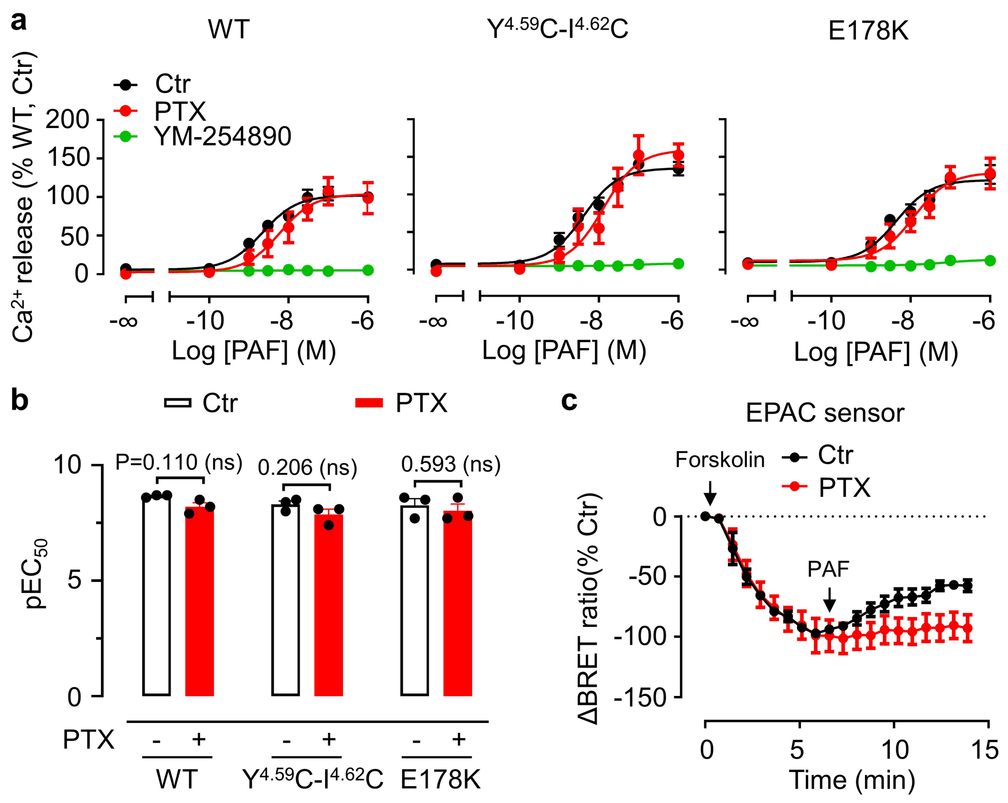

Supplementary Figure 13. (a) Intracellular Ca^{2+} responses mediated by the indicated receptors upon stimulation with PAF, after or without treatment with PTX (100 ng/ml) or YM-254890 (100 nM). **(b)** The indicated pEC_{50} efficacy is from the dose-response in panel *a*. **(c)** Monitoring cAMP mediated by PAF receptor after or without treatment with PTX (100 ng/ml). The exchange protein directly activated by cAMP (EPAC) BRET sensor was used to measure the cAMP in HEK-293 cells. Forskolin (10 μM) and PAF (5 μM) were used to stimulate the cells. Data are mean ± SEM from n=3 biologically independent experiments performed in triplicates and normalized to Ctr (cells not treated with PTX or YM-254890). Data are analyzed using unpaired two-tailed *t*-test.

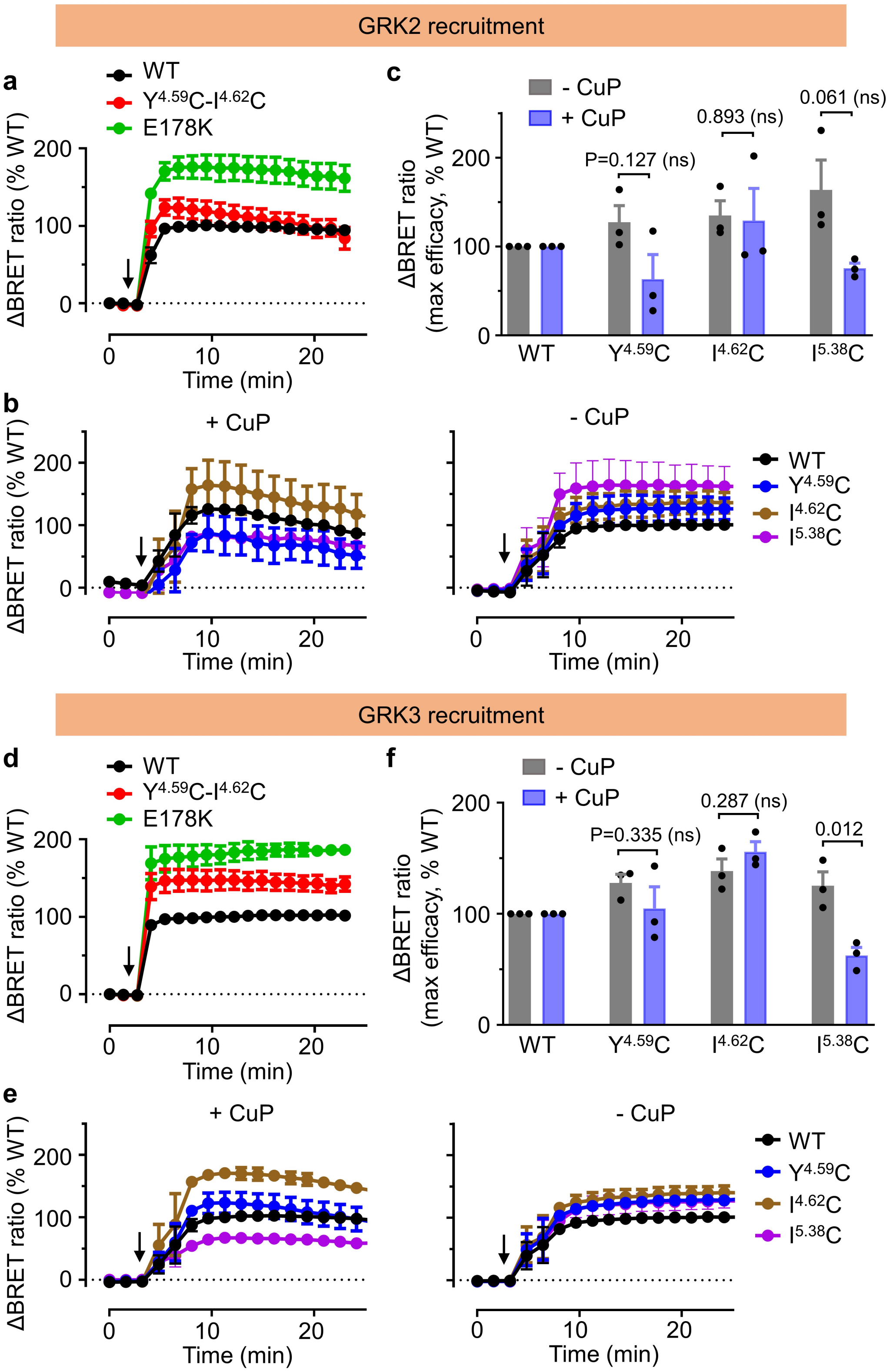

Supplementary Figure 14. Dimerization of PAFR does not impair GRK2 and GRK3 recruitment. **(a, d)** Kinetics of the BRET signal between the indicated Flag-tagged PAFR-Venus mutant and GRK2-Rluc *(a)* or GRK3-Rluc *(d)* after injection of PAF (1 μM, arrow), without treatment with CuP. **(b, e)** Kinetics of the BRET signal between the indicated Flag-tagged PAFR-Venus mutant and GRK2-Rluc or GRK3-Rluc after injection of PAF (1 μM,

arrow), after or without treatment with CuP. **(c, f)** Maximal efficacy of the BRET signals is from panels *b* and *e*, respectively. In *a-f*, data are mean ± SEM from n=3 biologically independent experiments performed in triplicates and normalized to WT, and analyzed using one-way ANOVA with Dunnett's multiple comparisons test.

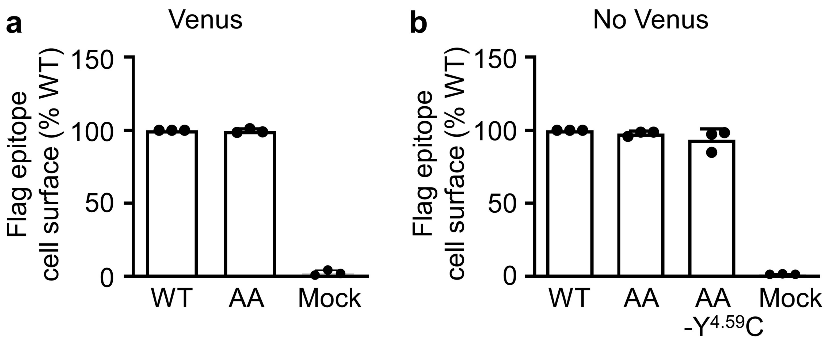

Supplementary Figure 15. Cell surface expression by ELISA assay of the Venus-tagged or none Venus-tagged PAFR mutant deficient for βarr recruitment (AA) containing or not the mutation $Y^{4.59}C$, and comparison to the WT. Data are mean ± SEM from n=3 biologically independent experiments performed in triplicates and normalized to WT.

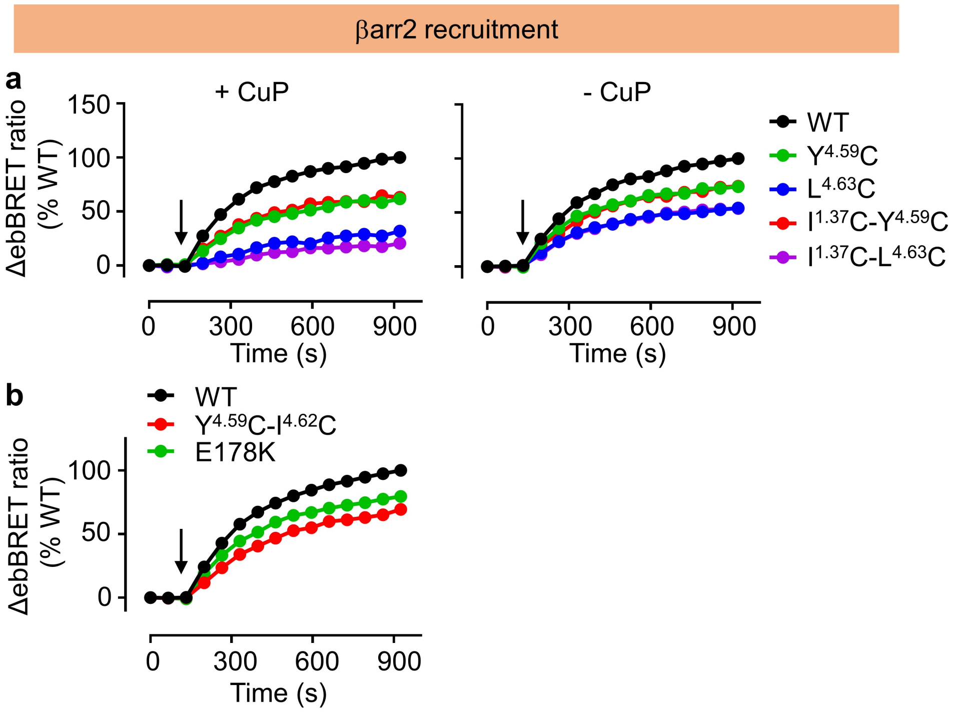

Supplementary Figure 16. Di/oligomerization of PAFR prevents βarr2 recruitment. (a) Kinetics of βarr2 recruitment to the cell membrane by the indicated Flag-tagged PAFR cysteine mutants, after or without treatment with CuP, upon stimulation with PAF (1 μM, arrow) and monitored by the variation of ebBRET signal between βarr2-Rluc and CAAX-Venus. **(b)** Kinetics of the BRET signal after injection of PAF (1 μM, arrow) for the indicated Flag-tagged PAFR cysteine mutant or genetic variant, without treatment with CuP. Data are mean ± SEM from n=3 biologically independent experiments performed in triplicates and normalized to WT.

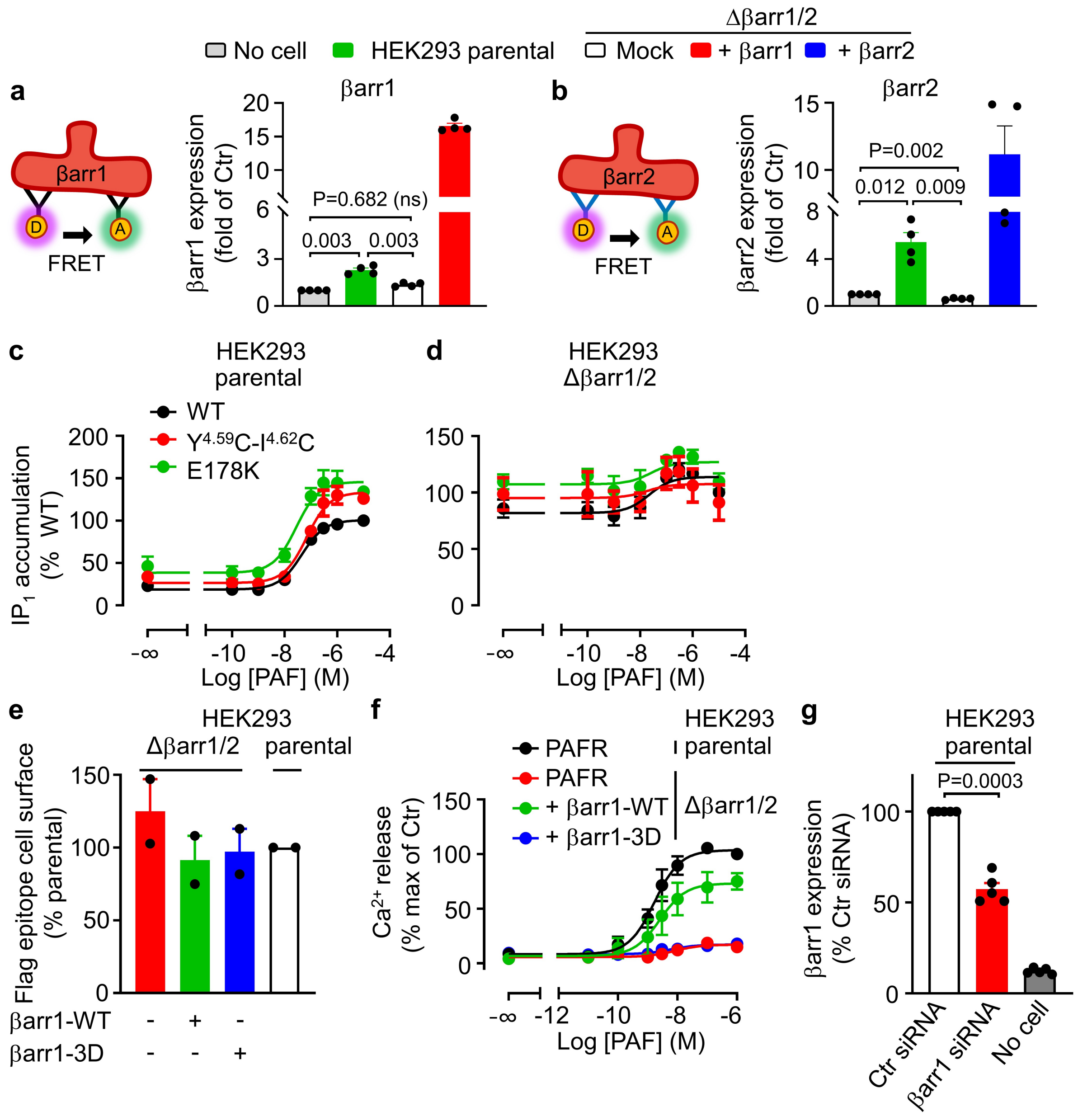

Supplementary Figure 17. (a,b) Scheme illustrating the TR-FRET based βarrs quantification assay, where two antibodies highly specific for two distinct epitopes on βarr1 or βarr2, one labeled with a donor fluorophore and the other with an acceptor are used. In the presence of βarr1 or βarr2 in a cell extract, the addition of these conjugates brings the donor fluorophore into close proximity with the acceptor and thereby generates a FRET signal. Expression of endogenous βarr1 or βarr2 in parental HEK-293, Δβarr1/2 HEK-293 cells (mock), and overexpressed βarr1 or βarr2 in Δβarr1/2 HEK-293 cells are the different conditions tested. Lysis buffer only (no cell, Ctr) was used as negative control. Data are mean ± SEM from n=4 biologically independent experiments performed in triplicates, normalized to Ctr and analyzed using unpaired two-tailed *t*-test. **(c, d)** IP_1 accumulation by the indicated PAFR mutants upon stimulation with PAF in parental (left) or Δβarr1/2 HEK-293 cells (right). Data are mean ±

SEM from n=3 biologically independent experiments performed in triplicates and normalized to WT. **(e)** Cell surface expression of WT PAFR in parental or Δβarr1/2 HEK-293 cells with or without co-transfection of βarr1-WT or βarr1-3D, by ELISA assay. Data are mean ± SEM from n=2 biologically independent experiments performed in triplicates and normalized to parental HEK293 cells. (**f**) Ca^{2+} release mediated by PAFR upon stimulation with PAF in parental (Ctr) or Δβarr1/2 HEK-293 cells. Data are mean ± SEM from n=3 biologically independent experiments performed in triplicates and normalized to Ctr. **(g)** Expression of endogenous βarr1 in parental HEK-293 cells after transfection of Ctr siRNA or βarr1 siRNA. Lysis buffer only (no cell, Ctr) was used as negative control. Data are mean ± SEM from n=5 biologically independent experiments performed in triplicates, normalized to Ctr siRNA and analyzed using unpaired two-tailed *t*-test.

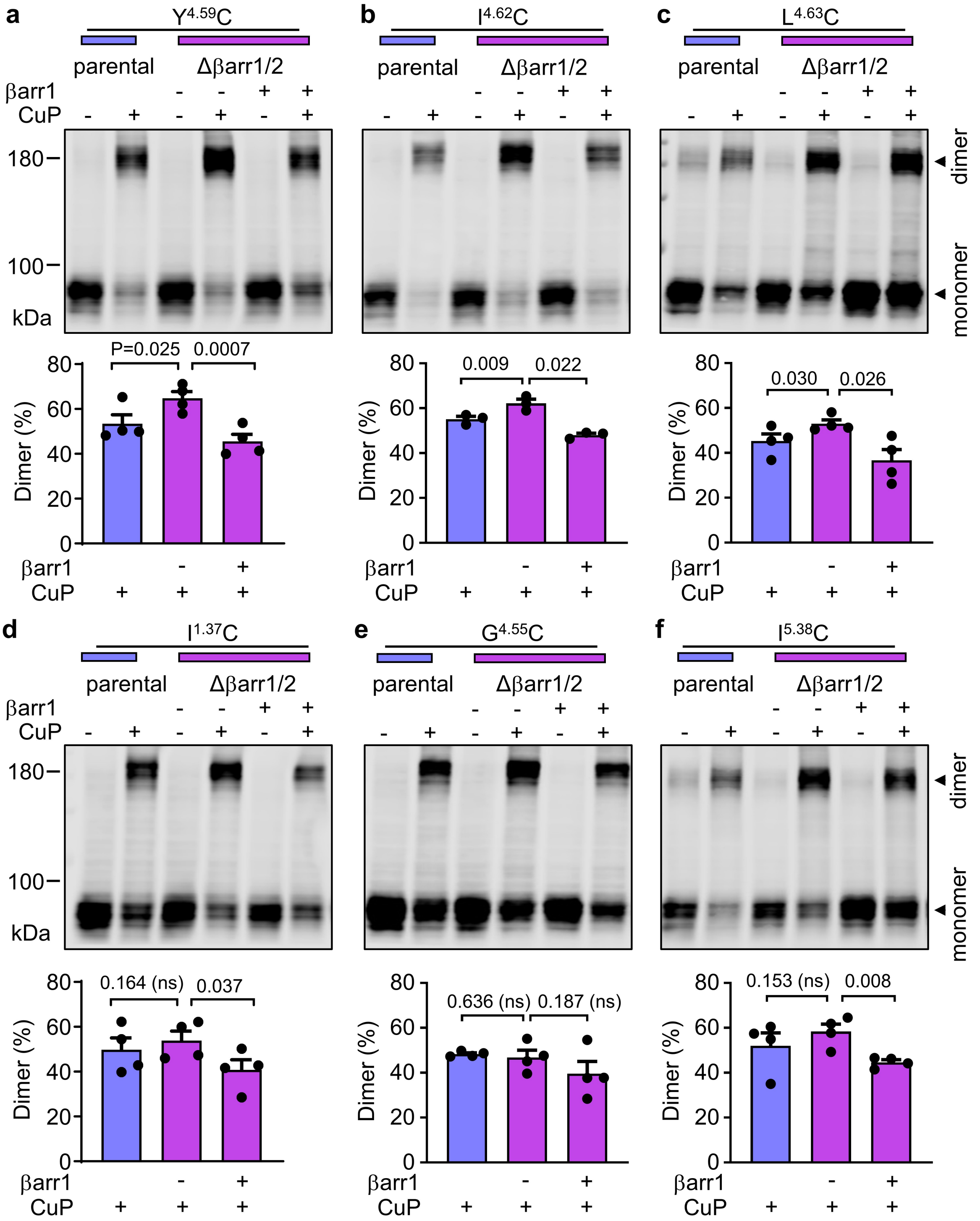

Supplementary Figure 18. β-arrestin impairs PAFR oligomers. (a-f) Blots showing cross-linking of cell surface Halo-PAFR subunits containing a cysteine substitution in TM1, TM4 or TM5 as indicated, with or without treatment with CuP in parental or Δβarr1/2 HEK-293 cells. Data are mean ± SEM from n=3-4 biologically independent experiments and analyzed using paired two-tailed *t*-test. Blot images are representative of a typical experiment from n=3-4 biologically independent experiments.

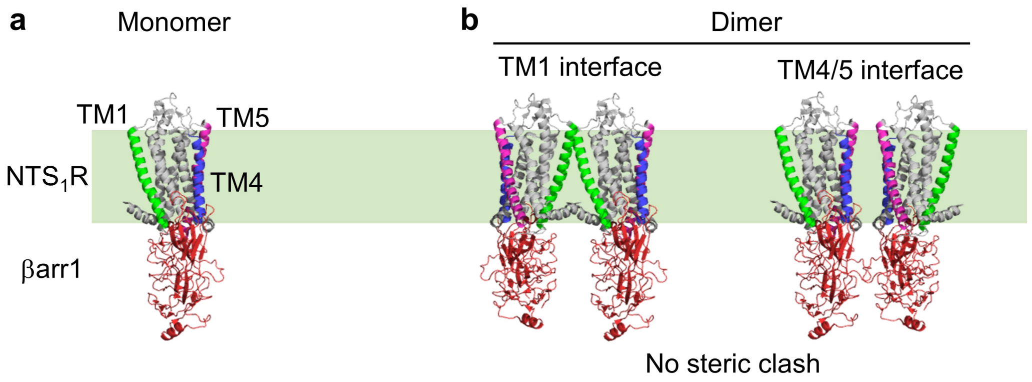

Supplementary Figure 19. (a) Structure of the complex between the neurotensin NTS1 receptor and βarr1 (PDB 6UP7). **(b)** 3D models of dimeric version of this structure either through the TM1 or TM4/5 interfaces, where no steric clash is observed between the two βarr1.

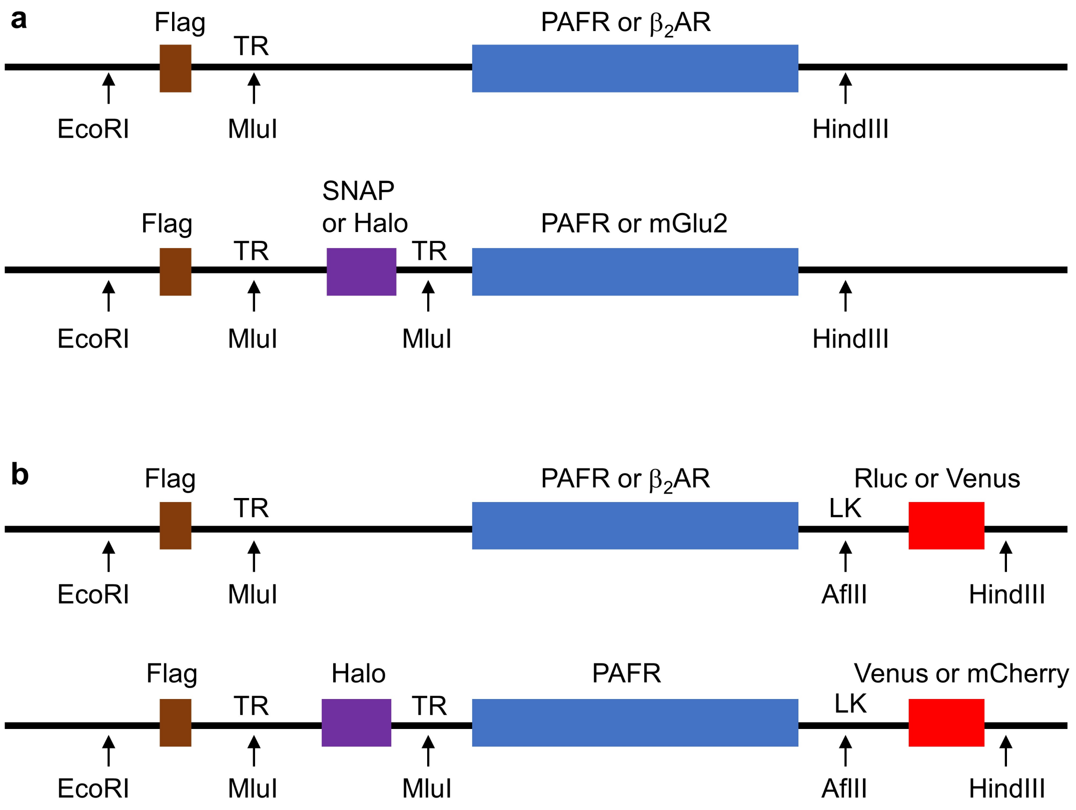

Supplementary Figure 20. Schematic representation of the constructs used for the PAF, β_2-adrenergic and mGlu2 receptors. TR and LK correspond to the linkers that encode for Thr-Arg and Leu-Lys, respectively.

Flag-PAFR

MVLLLILSVLLLKEDVRGSAQSTRPVDYKDDDDKTREPHDSSHMDSEFRYTLFPIVYSIIFVLGVIANGYVLWVFARLYPCKKFNEIKI
FMVNLTMADMLFLITLPLWIVYYQNQGNWILPKFLCNVAGCLFFINTYCSVAFLGVITYNRFQAVTRPIKTAQANTRKRGISLSLVIWV
AIVGAASYFLILDSTNTVPDSAGSGNVTRCFEHYEKGSVPVLIIHIFIVFSFFLVFLIILFCNLVIIRTLLMQPVQQQRNAEVKRRALWMV
CTVLAVFIICFVPHHVVQLPWTLAELGFQDSKFHQAINDAHQVTLCLLSTNCVLDPVIYCFLTKKFRKHLTEKFYSMRSSRKCSRATT
DTVTEVVVPFNQIPGNSLKN*

Flag-β_2AR

MVLLLILSVLLLKEDVRGSAQSTRPVDYKDDDDKTRGQPGNGSAFLLAPNGSHAPDHDVTQERDEVWVVGMGIVMSLIVLAIVFG
NVLVITAIAKFERLQTVTNYFITSLACADLVMGLAVVPFGAAHILMKMWTFGNFWCEFWTSIDVLCVTASIETLCVIAVDRYFAITSP
FKYQSLLTKNKARVIILMVWIVSGLTSFLPIQMHWYRATHQEAINCYANETCCDFFTNQAYAIASSIVSFYVPLVIMVFVYSRVFQE
AKRQLQKIDKSEGRFHVQNLSQVEQDGRTGHGLRRSSKFCLKEHKALKTLGIIMGTFTLCWLPFFIVNIVHVIQDNLIRKEVYILLN
WIGYVNSGFNPLIYCRSPDFRIAFQELLCLRRSSLKAYGNGYSSNGNTGEQSGYHVEQEKENKLLCEDLPGTEDFVGHQGTVPS
DNIDSQGRNCSTNDSLL*

Flag-SNAP-PAFR

MVLLLILSVLLLKEDVRGSAQSTRPVDYKDDDDKTRGSDKDCEMKRTTLDSPLGKLELSGCEQGLHEIKLLGKGTSAADAVEVPAP
AAVLGGPEPLMQATAWLNAYFHQPEAIEEFPVPALHHPVFQQESFTRQVLWKLLKVVKFGEVISYQQLAALAGNPAATAAVKTAL
SGNPVPILIPCHRVVSSSGAVGGYEGGLAVKEWLLAHEGHRLGKPGLGTREPHDSSHMDSEFRYTLFPIVYSIIFVLGVIANGYVL
WVFARLYPCKKFNEIKIFMVNLTMADMLFLITLPLWIVYYQNQGNWILPKFLCNVAGCLFFINTYCSVAFLGVITYNRFQAVTRPIKTA
QANTRKRGISLSLVIWVAIVGAASYFLILDSTNTVPDSAGSGNVTRCFEHYEKGSVPVLIIHIFIVFSFFLVFLIILFCNLVIIRTLLMQPV
QQQRNAEVKRRALWMVCTVLAVFIICFVPHHVVQLPWTLAELGFQDSKFHQAINDAHQVTLCLLSTNCVLDPVIYCFLTKKFRKHL
TEKFYSMRSSRKCSRATTDTVTEVVVPFNQIPGNSLKN*

Flag-SNAP-mGlu2

MVLLLILSVLLLKEDVRGSAQSTRPVDYKDDDDKTRGSDKDCEMKRTTLDSPLGKLELSGCEQGLHEIKLLGKGTSAADAVEVPAP
AAVLGGPEPLMQATAWLNAYFHQPEAIEEFPVPALHHPVFQQESFTRQVLWKLLKVVKFGEVISYQQLAALAGNPAATAAVKTAL
SGNPVPILIPCHRVVSSSGAVGGYEGGLAVKEWLLAHEGHRLGKPGLGTRKKVLTLEGDLVLGGLFPVHQKGGPAEECGPVNEH
RGIQRLEAMLFALDRINRDPHLLPGVRLGAHILDSCSKDTHALEQALDFVRASLSRGADGSRHICPDGSYATHSDAPTAVTGVIGG
SYSDVSIQVANLLRLFQIPQISYASTSAKLSDKSRYDYFARTVPPDFFQAKAMAEILRFFNWTYVSTVASEGDYGETGIEAFELEAR
ARNICVATSEKVGRAMSRAAFEGVVRALLQKPSARVAVLFTRSEDARELLAATQRLNASFTWVASDGWGALESVVAGSERAAEG
AITIELASYPISDFASYFQSLDPWNNSRNPWFREFWEERFHCSFRQRDCAAHSLRAVPFEQESKIMFVVNAVYAMAHALHNMHRA
LCPNTTHLCDAMRPVNGRRLYKDFVLNVKFDAPFRPADTDDEVRFDRFGDGIGRYNIFTYLRAGSGRYRYQKVGYWAEGLTLDT
SFIPWASPSAGPLPASRCSEPCLQNEVKSVQPGEVCCWLCIPCQPYEYRLDEFTCADCGLGYWPNASLTGCFELPQEYIRWGDA
WAVGPVTIACLGALATLFVLGVFVRHNATPVVKASGRELCYILLGGVFLCYCMTFVFIAKPSTAVCTLRRLGLGTAFSVCYSALLTK
TNRIARIFGGAREGAQRPRFISPASQVAICLALISGQLLIVAAWLVVEAPGTGKETAPERREVVTLRCNHRDASMLGSLAYNVLLIAL
CTLYAFKTRKCPENFNEAKFIGFTMYTTCIIWLAFLPIFYVTSSDYRVQTTTMCVSVSLSGSVVLGCLFAPKLHIILFQPQKNVVSHR
APTSRFGSAAPRASANLGQGSGSQFVPTVCNGREVVDSTTSSL*

Flag-Halo-PAFR

MVLLLILSVLLLKEDVRGSAQSTRPVDYKDDDDKTRAEIGTGFPFDPHYVEVLGERMHYVDVGPRDGTPVLFLHGNPTSSYVWRN
IIPHVAPTHRCIAPDLIGMGKSDKPDLGYFFDDHVRFMDAFIEALGLEEVVLVIHDWGSALGFHWAKRNPERVKGIAFMEFIRPIPT
WDEWPEFARETFQAFRTTDVGRKLIIDQNVFIEGTLPMGVVRPLTEVEMDHYREPFLNPVDREPLWRFPNELPIAGEPANIVALVE
EYMDWLHQSPVPKLLFWGTPGVLIPPAEAARLAKSLPNCKAVDIGPGLNLLQEDNPDLIGSEIARWLSTLEISGTREPHDSSHMDS
EFRYTLFPIVYSIIFVLGVIANGYVLWVFARLYPCKKFNEIKIFMVNLTMADMLFLITLPLWIVYYQNQGNWILPKFLCNVAGCLFFINT
YCSVAFLGVITYNRFQAVTRPIKTAQANTRKRGISLSLVIWVAIVGAASYFLILDSTNTVPDSAGSGNVTRCFEHYEKGSVPVLIIHIFI
VFSFFLVFLIILFCNLVIIRTLLMQPVQQQRNAEVKRRALWMVCTVLAVFIICFVPHHVVQLPWTLAELGFQDSKFHQAINDAHQVTL
CLLSTNCVLDPVIYCFLTKKFRKHLTEKFYSMRSSRKCSRATTDTVTEVVVPFNQIPGNSLKN*

Flag-Halo-mGlu2

MVLLLILSVLLLKEDVRGSAQSTRPVDYKDDDDKTRAEIGTGFPFDPHYVEVLGERMHYVDVGPRDGTPVLFLHGNPTSSYVWRN
IIPHVAPTHRCIAPDLIGMGKSDKPDLGYFFDDHVRFMDAFIEALGLEEVVLVIHDWGSALGFHWAKRNPERVKGIAFMEFIRPIPT
WDEWPEFARETFQAFRTTDVGRKLIIDQNVFIEGTLPMGVVRPLTEVEMDHYREPFLNPVDREPLWRFPNELPIAGEPANIVALVE
EYMDWLHQSPVPKLLFWGTPGVLIPPAEAARLAKSLPNCKAVDIGPGLNLLQEDNPDLIGSEIARWLSTLEISGTRKKVLTLEGDLV
LGGLFPVHQKGGPAEECGPVNEHRGIQRLEAMLFALDRINRDPHLLPGVRLGAHILDSCSKDTHALEQALDFVRASLSRGADGSR
HICPDGSYATHSDAPTAVTGVIGGSYSDVSIQVANLLRLFQIPQISYASTSAKLSDKSRYDYFARTVPPDFFQAKAMAEILRFFNWT
YVSTVASEGDYGETGIEAFELEARARNICVATSEKVGRAMSRAAFEGVVRALLQKPSARVAVLFTRSEDARELLAATQRLNASFT
WVASDGWGALESVVAGSERAAEGAITIELASYPISDFASYFQSLDPWNNSRNPWFREFWEERFHCSFRQRDCAAHSLRAVPFEQ
ESKIMFVVNAVYAMAHALHNMHRALCPNTTHLCDAMRPVNGRRLYKDFVLNVKFDAPFRPADTDDEVRFDRFGDGIGRYNIFTYL
RAGSGRYRYQKVGYWAEGLTLDTSFIPWASPSAGPLPASRCSEPCLQNEVKSVQPGEVCCWLCIPCQPYEYRLDEFTCADCGL
GYWPNASLTGCFELPQEYIRWGDAWAVGPVTIACLGALATLFVLGVFVRHNATPVVKASGRELCYILLGGVFLCYCMTFVFIAKPS
TAVCTLRRLGLGTAFSVCYSALLTKTNRIARIFGGAREGAQRPRFISPASQVAICLALISGQLLIVAAWLVVEAPGTGKETAPERREV
VTLRCNHRDASMLGSLAYNVLLIALCTLYAFKTRKCPENFNEAKFIGFTMYTTCIIWLAFLPIFYVTSSDYRVQTTTMCVSVSLSGSV
VLGCLFAPKLHIILFQPQKNVVSHRAPTSRFGSAAPRASANLGQGSGSQFVPTVCNGREVVDSTTSSL*

Supplementary Figure 21. Sequences of the constructs used for the PAF, β_2-adrenergic and mGlu2 receptors. The signal peptide of mGlu5 (black), Flag-tag (brown), SNAP-tag and Halo-tag (purple), amino acids linker (green), and receptors (blue) are indicated.

Flag-PAFR-Rluc

MVLLLILSVLLLKEDVRGSAQSTRPVDYKDDDDKTREPHDSSHMDSEFRYTLFPIVYSIIFVLGVIANGYVLWVFARLYPCKKFN
EIKIFMVNLTMADMLFLITLPLWIVYYQNQGNWILPKFLCNVAGCLFFINTYCSVAFLGVITYNRFQAVTRPIKTAQANTRKRGISL
SLVIWVAIVGAASYFLILDSTNTVPDSAGSGNVTRCFEHYEKGSVPVLIIHIFIVFSFFLVFLIILFCNLVIIRTLLMQPVQQQRNAEV
KRRALWMVCTVLAVFIICFVPHHVVQLPWTLAELGFQDSKFHQAINDAHQVTLCLLSTNCVLDPVIYCFLTKKFRKHLTEKFYS
MRSSRKCSRATTDTVTEVVVPFNQIPGNSLKNLKASKVYDPEQRKRMITGPQWWARCKQMNVLDSFINYYDSEKHAENAVIF
LHGNATSSYLWRHVVPHIEPVARCIIPDLIGMGKSGKSGNGSYRLLDHYKYLTAWFELLNLPKKIIFVGHDWGAALAFHYAYEH
QDRIKAIVHMESVVDVIESWDEWPDIEEDIALIKSEEGEKMVLENNFFVETVLPSKIMRKLEPEEFAAYLEPFKEKGEVRRPTLS
WPREIPLVKGGKPDVVQIVRNYNAYLRASDDLPKLFIESDPGFFSNAIVEGAKKFPNTEFVKVKGLHFLQEDAPDEMGKYIKSF
VERVLKNEQ*

Flag-β_2AR-Rluc

MVLLLILSVLLLKEDVRGSAQSTRPVDYKDDDDKTRGQPGNGSAFLLAPNGSHAPDHDVTQERDEVWVVGMGIVMSLIVLAIVF
GNVLVITAIAKFERLQTVTNYFITSLACADLVMGLAVVPFGAAHILMKMWTFGNFWCEFWTSIDVLCVTASIETLCVIAVDRYFAIT
SPFKYQSLLTKNKARVIILMVWIVSGLTSFLPIQMHWYRATHQEAINCYANETCCDFFTNQAYAIASSIVSFYVPLVIMVFVYSRVF
QEAKRQLQKIDKSEGRFHVQNLSQVEQDGRTGHGLRRSSKFCLKEHKALKTLGIIMGTFTLCWLPFFIVNIVHVIQDNLIRKEVYIL
LNWIGYVNSGFNPLIYCRSPDFRIAFQELLCLRRSSLKAYGNGYSSNGNTGEQSGYHVEQEKENKLLCEDLPGTEDFVGHQGT
VPSDNIDSQGRNCSTNDSLLLKASKVYDPEQRKRMITGPQWWARCKQMNVLDSFINYYDSEKHAENAVIFLHGNATSSYLWRH
VVPHIEPVARCIIPDLIGMGKSGKSGNGSYRLLDHYKYLTAWFELLNLPKKIIFVGHDWGAALAFHYAYEHQDRIKAIVHMESVVD
VIESWDEWPDIEEDIALIKSEEGEKMVLENNFFVETVLPSKIMRKLEPEEFAAYLEPFKEKGEVRRPTLSWPREIPLVKGGKPDVV
QIVRNYNAYLRASDDLPKLFIESDPGFFSNAIVEGAKKFPNTEFVKVKGLHFLQEDAPDEMGKYIKSFVERVLKNEQ*

Flag-PAFR-Venus

MVLLLILSVLLLKEDVRGSAQSTRPVDYKDDDDKTREPHDSSHMDSEFRYTLFPIVYSIIFVLGVIANGYVLWVFARLYPCKKFNE
IKIFMVNLTMADMLFLITLPLWIVYYQNQGNWILPKFLCNVAGCLFFINTYCSVAFLGVITYNRFQAVTRPIKTAQANTRKRGISLS
LVIWVAIVGAASYFLILDSTNTVPDSAGSGNVTRCFEHYEKGSVPVLIIHIFIVFSFFLVFLIILFCNLVIIRTLLMQPVQQQRNAEVK
RRALWMVCTVLAVFIICFVPHHVVQLPWTLAELGFQDSKFHQAINDAHQVTLCLLSTNCVLDPVIYCFLTKKFRKHLTEKFYSMR
SSRKCSRATTDTVTEVVVPFNQIPGNSLKNLKVSKGEELFTGVVPILVELDGDVNGHKFSVSGEGEGDATYGKLTLKLICTTGKL
PVPWPTLVTTLGYGLQCFARYPDHMKQHDFFKSAMPEGYVQERTIFFKDDGNYKTRAEVKFEGDTLVNRIELKGIDFKEDGNIL
GHKLEYNYNSHNVYITADKQKNGIKANFKIRHNIEDGGVQLADHYQQNTPIGDGPVLLPDNHYLSYQSALSKDPNEKRDHMVLL
EFVTAAGITLGMDELYK *

Flag-β_2AR-Venus

MVLLLILSVLLLKEDVRGSAQSTRPVDYKDDDDKTRGQPGNGSAFLLAPNGSHAPDHDVTQERDEVWVVGMGIVMSLIVLAIVF
GNVLVITAIAKFERLQTVTNYFITSLACADLVMGLAVVPFGAAHILMKMWTFGNFWCEFWTSIDVLCVTASIETLCVIAVDRYFAIT
SPFKYQSLLTKNKARVIILMVWIVSGLTSFLPIQMHWYRATHQEAINCYANETCCDFFTNQAYAIASSIVSFYVPLVIMVFVYSRV
FQEAKRQLQKIDKSEGRFHVQNLSQVEQDGRTGHGLRRSSKFCLKEHKALKTLGIIMGTFTLCWLPFFIVNIVHVIQDNLIRKEV
YILLNWIGYVNSGFNPLIYCRSPDFRIAFQELLCLRRSSLKAYGNGYSSNGNTGEQSGYHVEQEKENKLLCEDLPGTEDFVGHQ
GTVPSDNIDSQGRNCSTNDSLLLKVSKGEELFTGVVPILVELDGDVNGHKFSVSGEGEGDATYGKLTLKLICTTGKLPVPWPTL
VTTLGYGLQCFARYPDHMKQHDFFKSAMPEGYVQERTIFFKDDGNYKTRAEVKFEGDTLVNRIELKGIDFKEDGNILGHKLEYN
YNSHNVYITADKQKNGIKANFKIRHNIEDGGVQLADHYQQNTPIGDGPVLLPDNHYLSYQSALSKDPNEKRDHMVLLEFVTAAG
ITLGMDELYK *

Flag-Halo-PAFR-Venus

MVLLLILSVLLLKEDVRGSAQSTRPVDYKDDDDKTRAEIGTGFPFDPHYVEVLGERMHYVDVGPRDGTPVLFLHGNPTSSYVW
RNIIPHVAPTHRCIAPDLIGMGKSDKPDLGYFFDDHVRFMDAFIEALGLEEVVLVIHDWGSALGFHWAKRNPERVKGIAFMEFIRP
IPTWDEWPEFARETFQAFRTTDVGRKLIIDQNVFIEGTLPMGVVRPLTEVEMDHYREPFLNPVDREPLWRFPNELPIAGEPANIV
ALVEEYMDWLHQSPVPKLLFWGTPGVLIPPAEAARLAKSLPNCKAVDIGPGLNLLQEDNPDLIGSEIARWLSTLEISGTREPHDS
SHMDSEFRYTLFPIVYSIIFVLGVIANGYVLWVFARLYPCKKFNEIKIFMVNLTMADMLFLITLPLWIVYYQNQGNWILPKFLCNVA
GCLFFINTYCSVAFLGVITYNRFQAVTRPIKTAQANTRKRGISLSLVIWVAIVGAASYFLILDSTNTVPDSAGSGNVTRCFEHYEKG
SVPVLIIHIFIVFSFFLVFLIILFCNLVIIRTLLMQPVQQQRNAEVKRRALWMVCTVLAVFIICFVPHHVVQLPWTLAELGFQDSKFHQ
AINDAHQVTLCLLSTNCVLDPVIYCFLTKKFRKHLTEKFYSMRSSRKCSRATTDTVTEVVVPFNQIPGNSLKNLKVSKGEELFTGV
VPILVELDGDVNGHKFSVSGEGEGDATYGKLTLKLICTTGKLPVPWPTLVTTLGYGLQCFARYPDHMKQHDFFKSAMPEGYVQ
ERTIFFKDDGNYKTRAEVKFEGDTLVNRIELKGIDFKEDGNILGHKLEYNYNSHNVYITADKQKNGIKANFKIRHNIEDGGVQLAD
HYQQNTPIGDGPVLLPDNHYLSYQSALSKDPNEKRDHMVLLEFVTAAGITLGMDELYK*

Flag-Halo-PAFR-mCherry

MVLLLILSVLLLKEDVRGSAQSTRPVDYKDDDDKTRAEIGTGFPFDPHYVEVLGERMHYVDVGPRDGTPVLFLHGNPTSSYVWRNII
PHVAPTHRCIAPDLIGMGKSDKPDLGYFFDDHVRFMDAFIEALGLEEVVLVIHDWGSALGFHWAKRNPERVKGIAFMEFIRPIPTWD
EWPEFARETFQAFRTTDVGRKLIIDQNVFIEGTLPMGVVRPLTEVEMDHYREPFLNPVDREPLWRFPNELPIAGEPANIVALVEEYM
DWLHQSPVPKLLFWGTPGVLIPPAEAARLAKSLPNCKAVDIGPGLNLLQEDNPDLIGSEIARWLSTLEISGTREPHDSSHMDSEFRY
TLFPIVYSIIFVLGVIANGYVLWVFARLYPCKKFNEIKIFMVNLTMADMLFLITLPLWIVYYQNQGNWILPKFLCNVAGCLFFINTYCSVA
FLGVITYNRFQAVTRPIKTAQANTRKRGISLSLVIWVAIVGAASYFLILDSTNTVPDSAGSGNVTRCFEHYEKGSVPVLIIHIFIVFSFFL
VFLIILFCNLVIIRTLLMQPVQQQRNAEVKRRALWMVCTVLAVFIICFVPHHVVQLPWTLAELGFQDSKFHQAINDAHQVTLCLLSTNC
VLDPVIYCFLTKKFRKHLTEKFYSMRSSRKCSRATTDTVTEVVVPFNQIPGNSLKNLKVSKGEEDNMAIIKEFMRFKVHMEGSVNGH
EFEIEGEGEGRPYEGTQTAKLKVTKGGPLPFAWDILSPQFMYGSKAYVKHPADIPDYLKLSFPEGFNWERVMNFEDGGVVTVTQD
SSLQDGEFIYKVKLRGTNFPSDGPVMQCRTMGWEASTERMYPEDGALKGEIKQRLKLKDGGHYDAEVKTTYKAKKPVQLPGAYN
VDIKLDILSHNEDYTIVEQYERAEGRHSTGGMDELYK*

Supplementary Figure 22. Sequence of the constructs used for the PAF, β_2-adrenergic and mGlu2 receptors. The signal peptide of mGlu5 (black), Flag-tag (brown), SNAP-tag and Halo-tag (purple), amino acids linker (green), receptors (blue), and Rluc-tag, Venus-tag, mCherry-tag (red) are indicated.
