# Supplementary material for: Biased signaling due to oligomerization of the G protein-coupled platelet-activating factor receptor
Source: Nat Commun. 2022 Oct 26;13:6365. doi: 10.1038/s41467-022-34056-4 (PMC9606269; doi:10.1038/s41467-022-34056-4)
Supplement: Supplementary file 2 — Reporting Summary [file 41467_2022_34056_MOESM2_ESM.pdf]

nature portfolio

Corresponding author(s): Jean-Philippe Pin, Jianfeng Liu, Philippe Rondard and Wei Ji

Last updated by author(s): Sep 28, 2022

Reporting Summary

Nature Portfolio wishes to improve the reproducibility of the work that we publish. This form provides structure for consistency and transparency in reporting. For further information on Nature Portfolio policies, see our Editorial Policies and the Editorial Policy Checklist.

Statistics

For all statistical analyses, confirm that the following items are present in the figure legend, table legend, main text, or Methods section.

n/a	Confirmed	
☐	☒	The exact sample size (*n*) for each experimental group/condition, given as a discrete number and unit of measurement
☐	☒	A statement on whether measurements were taken from distinct samples or whether the same sample was measured repeatedly
☐	☒	The statistical test(s) used AND whether they are one- or two-sided<br>*Only common tests should be described solely by name; describe more complex techniques in the Methods section.*
☒	☐	A description of all covariates tested
☒	☐	A description of any assumptions or corrections, such as tests of normality and adjustment for multiple comparisons
☐	☒	A full description of the statistical parameters including central tendency (e.g. means) or other basic estimates (e.g. regression coefficient) AND variation (e.g. standard deviation) or associated estimates of uncertainty (e.g. confidence intervals)
☐	☒	For null hypothesis testing, the test statistic (e.g. *F*, *t*, *r*) with confidence intervals, effect sizes, degrees of freedom and *P* value noted<br>*Give P values as exact values whenever suitable.*
☒	☐	For Bayesian analysis, information on the choice of priors and Markov chain Monte Carlo settings
☒	☐	For hierarchical and complex designs, identification of the appropriate level for tests and full reporting of outcomes
☒	☐	Estimates of effect sizes (e.g. Cohen's *d*, Pearson's *r*), indicating how they were calculated

Our web collection on statistics for biologists contains articles on many of the points above.

Software and code

Policy information about availability of computer code

Data collection	TR-FRET data were acquired using PHERAstar (BMG LabTech);<br>Gel images were acquired using Odyssey infrared scanner (LI-COR Biosciences);<br>Calcium release data were acquired using a Flexstation 3 microplate reader or FLIPR Tetra (Molecular Devices);<br>BRET data were acquired using Mithras LB940 (Berthold Technologies);<br>Radioactivity binding data were acquired using a scintillation counter (Packard Instrument).
Data analysis	Data were plotted and statistically analyzed using GraphPad Prism (Version 9, GraphPad Software);<br>Fluorescent images were analyzed using ImageJ (Version 1.440, National Institutes of Health);<br>Single molecular imaging data were analyzed using MATLAB (Version 1.0.0.1, MathWorks).

For manuscripts utilizing custom algorithms or software that are central to the research but not yet described in published literature, software must be made available to editors and reviewers. We strongly encourage code deposition in a community repository (e.g. GitHub). See the Nature Portfolio guidelines for submitting code & software for further information.

Data

Policy information about availability of data

All manuscripts must include a data availability statement. This statement should provide the following information, where applicable:

- Accession codes, unique identifiers, or web links for publicly available datasets
- A description of any restrictions on data availability
- For clinical datasets or third party data, please ensure that the statement adheres to our policy

Data supporting the findings of this manuscript are available from the corresponding authors upon reasonable request. The source data underlying Figs. 1b-d, 2a-d,

3a-k, 4a-g, 5b-d, 6a-f, 7b-d, 8a-h and Supplementary Figs 1b, 2c, 3a-b, 4a-d, 6a-b, 7a-d, 8b-c, 9b-e, 10b-h, 11a-c, 12a-e, 13a-c, 14a-f, 15a-b, 16a-b, 17a-g, 18a-f are provided as a Source Data file.

Field-specific reporting

Please select the one below that is the best fit for your research. If you are not sure, read the appropriate sections before making your selection.

☒ Life sciences ☐ Behavioural & social sciences ☐ Ecological, evolutionary & environmental sciences

For a reference copy of the document with all sections, see nature.com/documents/nr-reporting-summary-flat.pdf

Life sciences study design

All studies must disclose on these points even when the disclosure is negative.

Sample size	We did not perform power analysis to predetermine sample sizes, but our sample size were determined based on obtaining large enough data points per condition to test for normality of distribution and perform appropriate statistical analysis.
Data exclusions	Some individual outliers were excluded from the analysis (in most cases, less than three points per 96-well plates were excluded).
Replication	Number of independent experiments and replicates are indicated in the legends to the figures.
Randomization	This study is entirely based on cellular experiments. Thus, no allocation into experimental groups was needed which could be randomized.
Blinding	Collection and analysis of TIRF Photo bleaching data were determined manually by one investigator and rescored blindly by another. All the other data, blinding was not needed because data was collected and objectively quantified via computer software.

Reporting for specific materials, systems and methods

We require information from authors about some types of materials, experimental systems and methods used in many studies. Here, indicate whether each material, system or method listed is relevant to your study. If you are not sure if a list item applies to your research, read the appropriate section before selecting a response.

Materials & experimental systems

n/a	Involved in the study	
☐	☒	Antibodies
☐	☒	Eukaryotic cell lines
☒	☐	Palaeontology and archaeology
☒	☐	Animals and other organisms
☒	☐	Human research participants
☒	☐	Clinical data
☒	☐	Dual use research of concern

Methods

n/a	Involved in the study	
☒	☐	ChIP-seq
☒	☐	Flow cytometry
☒	☐	MRI-based neuroimaging

Antibodies

Antibodies used	Monoclonal mouse anti-Flag antibody (F1804, Sigma-Aldirich);<br>Goat anti-mouse secondary antibody coupled to horseradish peroxidase (115-035-003, Jackson Immunoresearch).
Validation	The monoclonal mouse anti-Flag antibody (ELISA), F1804, Sigma-Aldirich. PMID: 18388862

Eukaryotic cell lines

Policy information about cell lines

Cell line source(s)	The human HEK-293 cell line (ATCC, CRL-1573), COS-7 cells (3111C0001CCC000033, National Infrastructure of Cell Line Resources, China) and HEK-293 cells with targeted deletion of ARRB1 and ARRB2 (β-arrestin1/2 knock out) were kind gifts from Dr. Asuka Inoue (Tohoku University, Sendai, Miyagi, Japan).
Authentication	The cell lines used were not further authenticated.
Mycoplasma contamination	The cell line was tested negative for mycoplasma contamination (each month).

Commonly misidentified lines<br>(See ICLAC register)	No commonly misidentified cell lines were used in this study.
